# US population norms for the EQ-5D-5L and comparison of norms from face-to-face and online samples

**DOI:** 10.1007/s11136-020-02650-y

**Published:** 2020-10-06

**Authors:** Ruixuan Jiang, M. F. Bas Janssen, A. Simon Pickard

**Affiliations:** 1 Center for Observational and Real-World Evidence, Merck, Kenilworth, NJ USA; 2 EuroQol Group, Rotterdam, The Netherlands; 3 Section Medical Psychology and Psychotherapy, Department of Psychiatry, Erasmus MC, Rotterdam, The Netherlands; 4 Department of Pharmacy Systems, Outcomes, and Policy, University of Illinois At Chicago College of Pharmacy, 833 S Wood St, Chicago, IL 60612 USA

**Keywords:** Population norms, Normative values, EQ-5D-5L, Reference values, Health-related quality of life, Patient reported outcomes, Online, Face-to-face

## Abstract

**Purpose:**

Normative scores (norms) allow for comparisons between population(s) of interest and the general population, which is useful for burden of disease studies and cost-effectiveness analysis. The primary aim of this study was to estimate US visual analogue scale (EQ VAS) and utility-based norms for the EQ-5D-5L using the face-to-face sample. The secondary aim was to compare norms estimated in the face-to-face and online populations.

**Methods:**

This study estimated population norms from two general population surveys: (a) face-to-face and (b) online. In these surveys, respondents provided their health state using the EQ-5D-5L health classifier and the EQ VAS. Descriptive statistics, including mean, standard deviation (SD), 95% confidence interval, and median for the 5L utility and EQ VAS were estimated for each sample and across relevant respondent characteristics to serve as the basis for US EQ-5D-5L norms

**Results:**

Face-to-face sample respondents (*n* = 1134) were representative of the US adult general population. In this sample, mean (SD) utility decreased with increasing age until age 45 or greater (age 45–54: 0.816 (0.249) age 55–64: 0.815 (0.243) age 65–74: 0.824 (0.217) age 75 + : 0.811 (0.218)). With increasing age, more problems were reported on all dimensions except anxiety/depression; a smaller proportion of respondents age 65 and older reported problems with anxiety/depression (23.8%) as compared to the youngest respondents (42.1%). Online (*n* = 2018) mean utility and EQ VAS values were consistently lower than the face-to-face sample.

**Conclusions:**

The availability of US EQ-5D-5L norms facilitates interpretation and understanding of general population and patient health.

**Electronic supplementary material:**

The online version of this article (10.1007/s11136-020-02650-y) contains supplementary material, which is available to authorized users.

## Introduction

The EQ-5D is the most widely used generic multi-attribute utility instrument in the world, and it has numerous applications in health care [1]. It is applied in a variety of research and clinical practice settings, such as clinical trials, cost-utility analysis (CUA), patient surveillance, and population health measurement [2–8]. The EQ-5D was developed as a brief, generic measure of health which includes a health state classifier that has five dimensions: mobility, self-care, usual activities, pain/discomfort, and anxiety/depression. The first version of the EQ-5D had 3 levels of health problems (EQ-5D-3L), and more recently in 2011, a more descriptively rich 5-level version (EQ-5D-5L) was introduced which describes 3125 health states (D^L^ = 5^5^ = 3125) [9, 10]. An index-based utility score can be generated from self-assessments using the descriptive system, by applying a value set based on societal preferences for EQ-5D health states. The value set is based on preference choice tasks elicited from the general population. In addition, the health state classifier is also accompanied by the visual analogue scale (EQ VAS), anchored at 0 for “the worst health you can imagine” and 100 for “the best health you can imagine” [9]. Thus, the EQ-5D-5L can provide two summary scores of health that can inform decision-making: the patient’s self-rating of health on the EQ VAS, and an index-based utility score. The latter is used to facilitate the generation of a common metric of health in the form of quality-adjusted life-years (QALYs) in CUA that can inform resource allocation across health care sectors.

Among the numerous applications of the EQ-5D-5L, a set of utility index and EQ VAS benchmark values for the general population, i.e., population reference data or population norms, are useful for comparing burden of disease and as normative reference values [11]. Patient or sample values can be compared to these benchmark values to determine how individuals or groups measure against the general population in decision models, evaluation of clinical programs, assessment of public health in large-scale applications, etc. [6, 11–23]. Although the use of the EQ-5D-5L continues to expand, the US does not yet have population norms for comparison. In 2019, an EQ-5D-5L value set for the US was developed [24]. It was developed utilizing the standardized international protocol recommended by the EuroQol Group and used preferences from an adult sample representative of the US general population. Apart from the valuation of EQ-5D-5L health states, these respondents also self-reported their own health using the EQ-5D-5L and the EQ VAS, allowing for estimation of US EQ-5D-5L population norms [24].

In the past, most studies reporting population norms have been based on data collected in-person as part of population health studies or using mail surveys [11, 12]. The standardized international protocol for EQ-5D-5L valuation studies was developed for a face-to-face, interviewer-assisted setting to ensure the respondent task comprehension and preserve higher data quality for the challenging preference choice tasks [25, 26]. The US EQ-5D-5L study used quota-based sampling based on age, gender, race, and ethnicity to ensure representativeness of the sample for the population and examined non-quota-based respondents characteristics like education to support comparability to the general population. A substudy was also conducted that sought to replicate the face-to-face protocol using online panels, applying the same quota-based sampling criteria [27, 28]. The online and face-to-face samples were dissimilar in terms of health, and the online sample was also less comparable to the general population more than the face-to-face sample in terms of non-quota-based characteristics. Thus, the face-to-face valuation study was selected as the primary source of population norms for the present study. However, as online data collection continues to gain relevance, there is value in understanding the differences between the two sources of respondents.

The primary aim of this study was to estimate EQ-5D-5L EQ VAS and index-based norms for the US general population using the data from the US valuation face-to-face study. A secondary aim was to compare face-to-face and online samples based on non-quota-based characteristics to understand the issues of generalizability with respect to mode of data collection.

## Methods

### Data sources

#### EQ-5D-5L face-to-face valuation study

The purpose of the EQ-5D-5L face-to-face valuation study was to elicit preferences for EQ-5D-5L health states from a representative sample of the US adult general population. This study was granted Institutional Review Board (IRB) exemption by the University of Illinois at Chicago IRB. Collected preferences were used as the basis for modeling the US EQ-5D-5L value set [24]. The face-to-face study followed the internationally standardized EQ-5D-5L valuation study protocol based on a robust body of evidence [25, 29–32]. Respondents were recruited using a variety of in-person, community, and online advertising methods. They were quota-sampled using age, gender, race, and ethnicity to match the most recently available US population parameters at the time. Respondents indicated informed consent to the interviewer prior to survey participation.

Eleven interviewers traveled around the United States between May and August 2017 to survey respondents in one-on-one sessions in six metropolitan areas: Chicago, Philadelphia, Phoenix, Birmingham, Seattle, and Denver [24]. Interview areas were chosen based on their representativeness of the general US population and to ensure data collection in each of the four US census regions: Northeast, Midwest, West, and South. Interview sessions occurred at several locations around each metropolitan area, including both city and suburban/rural areas. Each interview was conducted as a face-to-face, computer-assisted personal interview (CAPI). Surveys were completed in Spanish or English based on the respondent’s preferred language.

At the start of these surveys, respondents described their own health using the EQ-5D-5L visual analogue scale (EQ VAS) and the EQ-5D-5L descriptive system by indicating their level of problems on each of the 5 dimensions on the day of the survey (no, slight, moderate, severe, or extreme problems/unable to) [24]. Index-based utility scores for each respondent’s self-reported EQ-5D-5L health state was estimated by applying the US value set [24]. The US value set was based on the composite time trade-off (cTTO) preferences, and index values ranged from − 0.573 for the worst (55,555) to 1 for the best (11,111) EQ-5D-5L health state. Respondent self-reported EQ VAS values were also used to estimate general population norms.

#### EQ-5D-5L online valuation study

The EuroQol valuation study protocol and survey were also replicated in an online valuation substudy. The study was determined to be eligible for an IRB exemption by the Western Institutional Review Board. Respondents in the online study were recruited from established online survey panels using the same strata as the face-to-face study. Respondents indicated informed consent on the first survey screen prior to participation. The sequence and content of self-reported respondent characteristics, including EQ VAS and as an EQ-5D-5L health state, were the same between online and face-to-face surveys. Online respondents self-completed the survey without any interviewer supervision.

### Analyses

Norms for the utility index and EQ VAS were estimated using descriptive statistics, including mean, standard deviation, 95% confidence interval, and median. Proportions of respondents endorsing each level of severity for the five dimensions were also calculated. Norms were computed for the face-to-face and online samples separately. Characteristics important for informing population health, CUA, and clinical outcomes research were included as stratifying variables in the present analyses, including socio-demographic information (age, gender, race, ethnicity), and health (general health status, number of regular prescription medications) [33, 34]. Age was divided into seven age bands (18–24, 25–34, 35–44, 45–54, 55–64, 65–74, 75+).

During the surveys, respondents also self-reported diagnoses of certain illnesses, and the mean utility and EQ VAS values were estimated for each diagnosis. Only illnesses that were self-reported by more than 100 respondents were included in these analyses due to instability of the estimates with small sample sizes.

Utility and EQ VAS were analyzed as continuous variables. Statistical testing for differences in mean VAS or utility across groups was conducted using t-tests (gender, ethnicity, experience with illness) or ANOVA (age, race, general health status, number of prescription medications). Significance was designated at *p* < 0.05. All statistical analyses were completed in SAS 9.4 (Cary, NC, USA) The online and face-to-face norms were qualitatively compared.

## Results

### US EQ-5D-5L norms

One thousand one hundred and thirty-four adult respondents were recruited as part of the US face-to-face valuation study [24]. The sample was representative of the US general population for age, gender, race, and ethnicity (Table 1). Compared to the general US population, the face-to-face sample was more likely not to have child dependents under 18 and attained education greater than secondary school. There were no missing data for the EQ-5D-5L health states nor the VAS. Some covariates were missing for a single respondent who had to terminate the interview early. Five respondents identified as non-binary for gender, but norms could not be generated due to the limited sample size.

**Table 1:** Face-to-face and Online respondent characteristics as compared to the US general population

Characteristic	US general population (*n* = 327,167,439)	Face-to-face sample (*n* = 1134)	Online sample (*n* = 2018)
Age, mean (SD), *n* (%)		46.9 (18.1)	45.6 (15.5)
18–24	12.1%	107 (9.4)	133 (6.6)
25–34	17.9%	251 (22.1)	494 (24.5)
35–44	16.3%	182 (16.0)	385 (19.1)
45–54	16.4%	212 (18.7)	330 (16.4)
55–64	16.7%	159 (14.0)	386 (19.1)
65–74	12.0%	127 (11.2)	252 (12.5)
75+	6.1%	96 (8.5)	38 (1.9)
Gender, *n* (%)			
Male	48.3%	564 (49.7)	973 (48.2)
Female	51.4%	565 (49.8)	1041 (51.6)
Gender, other	0.3%	5 (0.4)	4 (0.20)
Race, *n* (%)			
White	65.5%	685 (60.4)	1570 (77.8)
Black	11.9%	152 (13.4)	258 (12.8)
Hispanic ethnicity, *n* (%)	15.0%	208 (18.3)	308 (15.3)
Education level greater than secondary, *n* (%)	58.9%	732 (64.6)	1316 (65.2)
Child dependents (may choose more than 1)			
None	71.2%	916 (80.8)	1377 (68.2)
Child(ren), ≤ 5 years old	–	68 (6.0)	221 (11.0)
Child(ren), 6 to 17 years old	–	180 (15.9)	536 (25.6)
Primary health insurance			
None	10.3%	98 (8.6)	211 (10.5)
Public	35.4%	480 (42.3)	734 (36.4)
Private	68.7%	555 (49.1)	1073 (53.2)
Country of birth, United States		983 (86.7)	1903 (94.3)
History of illness, *n* (%)			
Hypertension	32.0%	270 (23.8)	507 (25.1)
Arthritis	22.7%	267 (23.5)	445 (22.1)
Diabetes	9.4%	111 (9.8)	223 (11.1)
Heart failure	2.2%	20 (1.8)	28 (1.4)
Stroke	1.8–2.4%	23 (2.0)	39 (1.9)
Bronchitis	3.6%	29 (2.6)	50 (2.5)
Asthma	7.5%	132 (11.6)	195 (9.7)
Depression	25.7%	295 (26.0)	438 (21.7)
Migraine	16.0%	164 (14.5)	232 (11.5)
Cancer	5.9%	65 (5.7)	42 (2.1)
None	–	372 (32.8)	692 (34.3)
Health status, *n* (%)			
Excellent	20.0%	227 (20.0)	245 (12.1)
Very good	33.6%	421 (37.2)	695 (34.4)
Good	31.2%	332 (29.3)	730 (36.2)
Fair	12.5%	124 (10.9)	290 (14.4)
Poor	2.7%	29 (2.6)	58 (2.9)

Of the face-to-face sample, 31.2% of the respondents reported no problems on any of the EQ-5D-5L dimensions (11,111). (Table 2) The mean (standard deviation) utility value for the face-to-face sample was 0.851 (0.205). (Table 3) Mean utility differed across age groups (*p* < 0.001) and decreased with increasing age until the 45–54 age band. Means for age bands 45–54 and 55–64 were similar − 0.816 (0.249) and 0.815 (0.243), respectively. The mean increased for age band 65–74: 0.824 (0.217) and decreased again for the oldest age band of 75+ : 0.811 (0.218). Women and men had similar mean utility scores: 0.856 (0.191) versus 0.847 (0.219) (*p* = 0.487). No statistically significant differences in mean utility scores were identified across race and ethnicity categories.

**Table 2:** Most frequent self-reported EQ-5D-5L health states in the face-to-face sample (frequencies greater than or equal to 0.5%)

EQ-5D-5L health state	*N*	%	EQ-5D-5L health state	*N*	%
11111	354	31.2	21122	10	0.9
11121	138	12.2	11133	8	0.7
11112	95	8.4	11213	7	0.6
11122	64	5.6	11222	7	0.6
21121	37	3.3	21211	7	0.6
21111	24	2.1	21233	7	0.6
11123	19	1.7	11132	6	0.5
11113	18	1.6	11223	6	0.5
21222	17	1.5	11232	6	0.5
11221	14	1.2	21132	6	0.5
11131	12	1.1	21223	6	0.5
21221	12	1.1	31121	6	0.5
21231	12	1.1	31131	6	0.5
11211	11	1

**Table 3:** Face-to-face index and VAS-based norms by respondent characteristics

	*n*	%	US EQ-5D-5L Utility					VAS				
			Mean	Standard deviation	95% CI	Median	*P*-value	Mean	Standard deviation	95% CI	Median	*P*-value
Overall	1134	100	0.851	0.205	(0.839,0.863)	0.940		80.4	15.6	(79.5, 81.3)	85.0	
Age												
< 25	107	9.4	0.919	0.127	(0.894, 0.943)	0.943	< 0.001	84.9	11.8	(82.6, 87.1)	90.0	< 0.001
25–34	251	22.1	0.911	0.111	(0.897, 0.925)	0.940		84.4	10.4	(83.1, 85.7)	85.0	
35–44	182	16.0	0.841	0.210	(0.811, 0.872)	0.932		78.1	15.4	(75.9, 80.4)	80.0	
45–54	212	18.7	0.816	0.249	(0.782, 0.85)	0.904		75.9	18.6	(73.4, 78.4)	80.0	
55–64	159	14.0	0.815	0.243	(0.777, 0.853)	0.940		78.8	18.8	(75.9, 81.8)	80.0	
65–74	127	11.2	0.824	0.217	(0.786, 0.862)	0.904		80.7	15.1	(78.1, 83.4)	85.0	
75+	96	8.5	0.811	0.218	(0.767, 0.855)	0.858		81.1	15.6	(78, 84.3)	85.0	
Gender												
Male	564	50.0	0.847	0.219	(0.829, 0.865)	0.940	0.487	79.8	16.4	(42.7, 91.3)	82.5	0.148
Female	565	50.0	0.856	0.191	(0.840, 0.872)	0.940		81.1	14.7	(78.4, 81.1)	85.0	
Race category												
White	685	60.4	0.849	0.199	(0.834, 0.864)	0.940	0.550	81.0	14.5	(79.9, 82.1)	85.0	0.260
Black	152	13.4	0.840	0.207	(0.807, 0.873)	0.902		79.4	16.2	(76.8, 82)	80.0	
Other	297	26.2	0.861	0.220	(0.836, 0.886)	0.940		79.5	17.7	(77.4,81.5)	85.0	
Ethnicity												
Hispanic	208	18.3	0.843	0.226	(0.812, 0.874)	0.940	0.544	78.9	17.7	(76.5, 81.3)	82.5	0.136
Not Hispanic	926	81.7	0.853	0.200	(0.840, 0.866)	0.940		80.7	15.1	(79.7, 81.7)	85.0	
General health												
Excellent	227	20.0	0.951	0.096	(0.939, 0.964)	1.000	< 0.001	91.7	9.3	(90.4, 92.9)	92.0	< 0.001
Very good	421	37.2	0.910	0.131	(0.897, 0.922)	0.940		85.0	8.6	(84.2, 85.8)	85.0	
Good	332	29.3	0.835	0.160	(0.818, 0.852)	0.883		76.5	13.0	(75.1, 77.9)	80.0	
Fair	124	10.9	0.632	0.262	(0.585, 0.678)	0.691		62.4	18.3	(59.1, 65.6)	60.0	
Poor	29	2.6	0.338	0.380	(0.194, 0.483)	0.385		46.3	21.0	(38.4, 54.3)	50.0	
Regular prescription medications												
0	461	40.7	0.910	0.154	(0.896, 0.924)	0.943	< 0.001	84.6	12.5	(83.5,85.7)	90.0	< 0.001
1	210	18.5	0.885	0.168	(0.862, 0.908)	0.940		82.4	14.3	(80.4,84.3)	85.0	
2–4	286	25.2	0.830	0.192	(0.808, 0.853)	0.883		78.7	15.8	(76.8,80.5)	80.0	
5 or more	176	15.5	0.691	0.283	(0.649, 0.733)	0.777		69.7	18.7	(66.9,72.5)	70.0	
Personal experience with serious illness												
No	665	58.6	0.903	0.143	(0.892, 0.914)	0.943	< 0.001	84.0	12.5	(83, 84.9)	86.0	< 0.001
Yes	469	41.4	0.777	0.253	(0.754, 0.800)	0.847		75.3	18.1	(73.6, 76.9)	80.0	
Family experience with serious illness												
No	177	15.6	0.888	0.175	(0.862, 0.914)	0.943	0.009	82.5	15.8	(80.1, 84.8)	86.0	0.054
Yes	957	84.4	0.844	0.210	(0.831, 0.858)	0.932		80.0	15.6	(79, 81)	85.0	
Experience caring for someone with serious illness												
No	498	43.9	0.878	0.178	(0.862, 0.893)	0.940	< 0.001	81.8	15.2	(80.5, 83.1)	85.0	0.007
Yes	636	56.1	0.830	0.222	(0.813, 0.848)	0.902		79.3	15.9	(78, 80.5)	80.0	
Health condition												
Arthritis	267	23.6	0.712	0.266	(0.680, 0.744)	0.777		74.2	17.8	(72.1, 76.4)	80.0	
Asthma	132	11.7	0.771	0.252	(0.728, 0.815)	0.836		76.1	15.9	(73.4, 78.9)	80.0	
Depression	295	26.0	0.708	0.258	(0.678, 0.738)	0.780		71.6	17.3	(69.6, 73.6)	75.0	
DM	111	9.8	0.788	0.233	(0.744, 0.832)	0.872		74.1	17.8	(70.8, 77.5)	80.0	
Hay fever	136	12.0	0.785	0.249	(0.743, 0.828)	0.858		77.2	15.2	(74.6, 79.8)	80.0	
Hypertension	270	23.8	0.793	0.223	(0.767, 0.820)	0.844		75.9	17.5	(73.8, 78.0)	80.0	
Migraine	164	14.5	0.774	0.240	(0.737, 0.811)	0.845		75.7	17.4	(73.0, 78.4)	80.0	
Sinusitis	114	10.1	0.730	0.279	(0.679, 0.782)	0.817		74.2	17.3	(71.0, 77.4)	80.0	

Mean utility scores decreased with poorer general health as respondents with excellent, very good, good, fair, and poor health had mean (SD) scores of 0.951 (0.096), 0.910 (0.131), 0.835 (0.160), 0.632 (0.262), and 0.338 (0.380), respectively. (Table 3) The mean utility also decreased with increasing number of regular prescriptions taken (*p* < 0.001). Respondents with experience with serious illness, whether personal, intra-familial, or caring for others, had lower mean utility and EQ VAS than those with without the experience (*p* < 0.01 for all comparisons).

The mean (SD) EQ VAS for the sample was 80.4 (15.6). (Table 3) The mean EQ VAS pattern across age bands differed from mean index. EQ VAS decreased with increasing age until the 45–54 age band and reached a nadir of 75.9 (18.6); it then increased through the rest of the age bands, reaching 81.1 (15.6) for respondents 75 and older. Across other respondent characteristics, VAS norm trends were comparable to those observed for utility norms.

Of the EQ-5D-5L dimensions, fewest respondents reported problems with self-care (6.5% of respondents) and the most respondents reported any problems with pain/discomfort (51% of respondents). (Table 4) For both the index score and EQ VAS-based norms across the entire sample, means generally decreased with increasing problems on each dimension. Few respondents endorsed severe (level 4) and extreme problems/unable to (level 5) on most dimensions. The prevalence of any problems on each EQ-5D-5L dimension increased for all dimensions with advancing age except for anxiety/depression, which had the opposite trend. (Supplementary material Appendix A).

**Table 4:** Face-to-face index and VAS-based norms by EQ-5D-5L dimension levels and gender

	Total						Men							Women					
	*n*	%	Mean	SD	95% CI	Median	*n*	%	Mean	SD	95% CI	Median		*n*	%	Mean	SD	95% CI	Median
US EQ-5D-5L utility																			
Mobility																			
1	812	71.6	0.936	0.086	(0.930, 0.942)	0.943	404	71.6	0.934	0.092	(0.925, 0.943)	0.943		405	71.7	0.939	0.077	(0.931, 0.946)	0.943
2	208	18.3	0.720	0.171	(0.696, 0.743)	0.749	99	17.6	0.736	0.176	(0.701, 0.772)	0.776		107	18.9	0.704	0.166	(0.672, 0.735)	0.738
3	79	7.0	0.624	0.198	(0.580, 0.668)	0.670	37	6.6	0.609	0.229	(0.532, 0.685)	0.670		42	7.4	0.638	0.168	(0.586, 0.690)	0.670
4	31	2.7	0.192	0.302	(0.081, 0.302)	0.224	20	3.5	0.229	0.289	(0.093, 0.364)	0.243		11	1.9	0.125	0.328	(− 0.095, 0.345)	0.198
5	4	0.4	0.115	0.497	(− 0.676, 0.905)	0.215	4	0.7	0.115	0.497	0.676, 0.905)	0.215							
Self-care																			
1	1060	93.5	0.885	0.144	(0.876, 0.893)	0.940	525	93.1	0.885	0.151	(0.872, 0.898)	0.940		530	93.8	0.886	0.137	(0.874, 0.897)	0.940
2	42	3.7	0.518	0.187	(0.460, 0.576)	0.533	18	3.2	0.533	0.197	(0.435, 0.631)	0.549		24	4.2	0.507	0.182	(0.430, 0.583)	0.532
3	25	2.2	0.264	0.299	(0.140, 0.387)	0.295	16	2.8	0.246	0.285	(0.094, 0.398)	0.209		9	1.6	0.295	0.337	(0.036, 0.555)	0.334
4	5	0.4	− 0.184	0.209	(− 0.443, 0.075)	− 0.183	3	0.5	− 0.096	0.202	(− 0.599, 0.406)	0.001		2	0.4	− 0.315	0.187	(− 1.992, 1.362)	− 0.315
5	2	0.2	− 0.017	0.756	(− 6.808, 6.775)	− 0.017	2	0.4	− 0.017	0.756	(− 6.808, 6.775)	− 0.017							
Usual activities																			
1	854	75.3	0.929	0.091	(0.923, 0.935)	0.943	436	77.3	0.926	0.095	(0.917, 0.935)	0.943		416	73.6	0.933	0.086	(0.924, 0.941)	0.943
2	178	15.7	0.720	0.149	(0.698, 0.742)	0.738	77	13.7	0.707	0.169	(0.668, 0.745)	0.745		98	17.3	0.732	0.132	(0.705, 0.758)	0.738
3	80	7.1	0.528	0.226	(0.478, 0.578)	0.582	40	7.1	0.486	0.258	(0.403, 0.568)	0.561		40	7.1	0.570	0.183	(0.512, 0.628)	0.582
4	16	1.4	0.051	0.240	(− 0.077, 0.179)	− 0.052	8	1.4	0.013	0.241	(− 0.189, 0.214)	− 0.083		8	1.4	0.089	0.248	(− 0.119, 0.296)	0.040
5	6	0.5	0.069	0.514	(− 0.470, 0.608)	0.113	3	0.5	0.041	0.655	(− 1.586, 1.669)	− 0.070		3	0.5	0.097	0.477	(− 1.088, 1.283)	0.295
Pain/discomfort																			
1	556	49.0	0.961	0.070	(0.955, 0.967)	1.000	272	48.2	0.962	0.071	(0.953, 0.970)	1.000		283	50.1	0.960	0.070	(0.952, 0.968)	1.000
2	374	33.0	0.853	0.096	(0.843, 0.863)	0.883	188	33.3	0.854	0.098	(0.840, 0.868)	0.883		183	32.4	0.853	0.095	(0.839, 0.867)	0.883
3	151	13.3	0.677	0.146	(0.653, 0.700)	0.705	72	12.8	0.695	0.157	(0.658, 0.732)	0.738		78	13.8	0.662	0.134	(0.631, 0.692)	0.683
4	39	3.4	0.259	0.206	(0.192, 0.325)	0.282	24	4.3	0.234	0.230	(0.137, 0.331)	0.228		15	2.7	0.298	0.158	(0.210, 0.386)	0.336
5	14	1.2	− 0.019	0.302	(− 0.193, 0.156)	− 0.070	8	1.4	− 0.002	0.327	(− 0.275, 0.272)	− 0.067		6	1.1	− 0.041	0.294	(− 0.349, 0.268)	− 0.103
Anxiety/depression																			
1	699	61.6	0.926	0.117	(0.918, 0.935)	1.000	357	63.3	0.923	0.126	(0.910, 0.936)	1.000		342	60.5	0.929	0.108	(0.918, 0.941)	1.000
2	272	24.0	0.820	0.177	(0.799, 0.841)	0.883	132	23.4	0.810	0.183	(0.778, 0.841)	0.883		137	24.2	0.829	0.173	(0.800, 0.858)	0.883
3	131	11.6	0.649	0.222	(0.611, 0.688)	0.716	56	9.9	0.673	0.215	(0.615, 0.730)	0.735		74	13.1	0.630	0.228	(0.577, 0.683)	0.683
4	24	2.1	0.331	0.356	(0.181, 0.482)	0.464	13	2.3	0.146	0.376	(− 0.081, 0.373)	0.158		10	1.8	0.552	0.165	(0.434, 0.670)	0.619
5	8	0.7	0.213	0.371	(− 0.097, 0.523)	0.278	6	1.1	0.295	0.303	(− 0.024, 0.613)	0.313		2	0.4	− 0.032	0.588	(− 5.311, 5.248)	− 0.032
Vas																			
Mobility																			
1	812	71.6	84.7	11.2	(83.9, 85.4)	87.0	404	71.6	84.3	11.1	(83.2, 85.4)	85.0		405	71.7	85.2	11.1	(84.1, 86.3)	90.0
2	208	18.3	74.6	16.6	(72.3, 76.8)	80.0	99	17.6	75.3	16.6	(72.0, 78.6)	80.0		107	18.9	74.1	16.7	(70.9, 77.3)	80.0
3	79	7.0%	67.7	16.8	(63.9, 71.4)	70.0	37	6.6	67.8	17.8	(61.8, 73.7)	70.0		42	7.4	67.6	16.1	(62.6, 72.6)	70.0
4	31	2.7	41.7	21.1	(34.0, 49.5)	40.0	20	3.5	36.2	21.7	(26.0, 46.4)	40.0		11	1.9	51.8	16.5	(40.8, 62.9)	50.0
5	4	0.4	64.3	12.0	(45.1, 83.4)	64.5	4	0.7	64.3	12.0	(45.1, 83.4)	64.5							
Self-care																			
1	1060	93.5	82.0	13.9	(81.2, 82.8)	85.0	525	93.1	81.6	14.2	(80.3, 82.8)	85.0		530	93.8	82.6	13.4	(81.4, 83.7)	85.0
2	42	3.7	63.8	14.3	(59.3, 68.2)	60.0	18	3.2	67.7	13.0	(61.2, 74.2)	70.0		24	4.2	60.8	14.8	(54.6, 67.1)	60.0
3	25	2.2	48.4	23.0	(38.9, 57.9)	49.0	16	2.8	44.9	24.8	(31.7, 58.1)	44.5		9	1.6	54.4	19.4	(39.5, 69.4)	60.0
4	5	0.4	42.4	30.7	(4.3, 80.5)	50.0	3	0.5	34.0	40.0	(− 65.2, 133.2)	24.0		2	0.4	55.0	7.1	(− 8.5, 118.5)	55.0
5	2	0.2	64.5	6.4	(7.3, 121.7)	64.5	2	0.4	64.5	6.4	(7.3, 121.7)	64.5							
Usual activities																			
1	854	75.3	84.6	11.9	(83.8, 85.4)	88.5	436	77.3	83.9	12.3	(82.7, 85.0)	85.0		416	73.6	85.3	11.4	(84.2, 86.4)	90.0
2	178	15.7	72.4	15.9	(70.0, 74.7)	75.0	77	13.7	71.0	16.8	(67.2, 74.8)	75.0		98	17.3	73.7	15.0	(70.7, 76.7)	77.5
3	80	7.1	61.2	18.0	(57.2, 65.2)	60.0	40	7.1	60.1	20.4	(53.5, 66.6)	67.5		40	7.1	62.4	15.3	(57.5, 67.3)	60.0
4	16	1.4	49.6	25.7	(35.9, 63.3)	50.0	8	1.4	47.5	33.3	(19.7, 75.3)	45.0		8	1.4	51.8	17.2	(37.4, 66.1)	55.0
5	6	0.5	59.8	21.8	(36.9, 82.7)	69.5	3	0.5	56.3	31.9	(− 23.0, 135.7)	69.0		3	0.5	63.3	11.5	(34.6, 92.0)	70.0
Pain/discomfort																			
1	556	49.0	86.1	11.4	(85.2, 87.1)	90.0	272	48.2	85.2	11.9	(83.8, 86.6)	90.0		283	50.1	87.0	10.8	(85.8, 88.3)	90.0
2	374	33.0%	80.1	13.6	(78.8, 81.5)	80.0	188	33.3	80.7	13.7	(78.7, 82.7)	80.0		183	32.4	79.8	13.4	(77.8, 81.7)	80.0
3	151	13.3	69.7	15.7	(67.1, 72.2)	70.0	72	12.8	70.1	15.8	(66.4, 73.8)	70.0		78	13.8	69.6	15.4	(66.1, 73.1)	70.0
4	39	3.4	53.1	20.4	(46.5, 59.7)	60.0	24	4.3	51.3	24.2	(41.1, 61.5)	55.0		15	2.7	56.0	12.6	(49.0, 63.0)	60.0
5	14	1.2	49.6	20.9	(37.5, 61.6)	50.0	8	1.4	46.1	23.7	(26.3, 66.0)	45.0		6	1.1	54.2	17.4	(35.9, 72.5)	55.0
Anxiety/depression																			
1	699	61.6	84.0	13.3	(83.0, 85.0)	90.0	357	63.3	83.2	14.2	(81.7, 84.6)	85.0		342	60.5	84.8	12.3	(83.5, 86.1)	90.0
2	272	24.0	79.1	14.7	(77.3, 80.9)	80.0	132	23.4	78.6	14.6	(76.1, 81.1)	80.0		137	24.2	79.7	14.8	(77.2, 82.2)	85.0
3	131	11.6	70.0	15.5	(67.3, 72.7)	70.0	56	9.9	71.0	15.3	(66.9, 75.1)	70.0		74	13.1	69.2	15.8	(65.5, 72.8)	70.0
4	24	2.1	58.1	25.2	(47.4, 68.7)	60.0	13	2.3	51.5	28.3	(34.4, 68.5)	60.0		10	1.8	68.5	18.4	(55.3, 81.7)	70.0
5	8	0.7	46.3	23.7	(26.4, 66.1)	42.5	6	1.1	45.8	28.0	(16.4, 75.2)	40.0		2	0.4	47.5	3.5	(15.7, 79.3)	47.5

Norms were also estimated separately by gender with additional stratification by age and general health status. (Table 5) Across age bands, women had higher mean index than men except for the 45–54 and 65–74 age bands where the pattern was reversed (45–54: mean (SD) men: 0.825 (0.256) women: 0.807 (0.241); 65–74: men: 0.827 (0.216) women: 0.821 (0.221)). The mean index and EQ VAS in both genders consistently decreased with decreasing general health. Men with self-reported excellent health had mean (SD) utility index of 0.942 (0.121), and those with poor health had 0.258 (0.344). Mean EQ VAS in men ranged from 91.2 (10.2) to 36.9 (19.7) by general health status. Women with self-reported excellent health had mean utility of 0.962 (0.057) whereas those with poor health had mean utility of 0.396 (0.412). Mean EQ VAS in women ranged from 92.2 (8.2) to 56.5 (18.6). Notably, women with poor health reported higher utility and EQ VAS than men with the same general health status.

**Table 5:** Face-to-face index and VAS-based norms by age and general health status stratified by gender

	Men						Women					
	*n*	%	Mean	Standard deviation	95% CI	Median	*n*	%	Mean	Standard deviation	95% CI	Median
EQ-5D-5L index												
Overall	564	50.0	0.847	0.219	(0.829, 0.865)	0.940	565	50.0	0.856	0.191	(0.840, 0.872)	0.940
Age												
< 25	54	9.6	0.906	0.157	(0.863, 0.949)	0.943	53	9.4	0.931	0.085	(0.908, 0.955)	0.943
25–34	118	20.9	0.907	0.114	(0.887, 0.928)	0.940	130	23.0	0.916	0.107	(0.897, 0.934)	0.943
35–44	86	15.2	0.841	0.212	(0.795, 0.886)	0.903	95	16.8	0.845	0.209	(0.803, 0.888)	0.940
45–54	110	19.5	0.825	0.256	(0.777, 0.873)	0.940	102	18.1	0.807	0.241	(0.759, 0.854)	0.883
55–64	91	16.1	0.807	0.266	(0.752, 0.863)	0.904	67	11.9	0.827	0.211	(0.776, 0.879)	0.940
65–74	70	12.4	0.827	0.216	(0.775, 0.878)	0.889	57	10.1	0.821	0.221	(0.762, 0.879)	0.904
75+	35	6.2	0.786	0.272	(0.693, 0.88)	0.818	61	10.8	0.825	0.181	(0.779, 0.872)	0.878
General health												
Excellent	118	20.9	0.942	0.121	(0.920, 0.964)	1.000	109	19.3	0.961	0.057	(0.950, 0.972)	1.000
Very good	208	36.9	0.902	0.155	(0.881, 0.923)	0.940	212	37.6	0.917	0.103	(0.903, 0.931)	0.940
Good	161	28.5	0.842	0.164	(0.816, 0.867)	0.883	169	30.0	0.830	0.158	(0.806, 0.854)	0.878
Fair	62	11.0	0.641	0.279	(0.570, 0.712)	0.718	61	10.8	0.623	0.247	(0.560, 0.687)	0.688
Poor	15	2.7	0.258	0.344	(0.068, 0.449)	0.219	13	2.3	0.396	0.412	(0.147, 0.645)	0.526
EQ VAS												
Overall	564	50.0	79.8	16.4	(78.4, 81.1)	82.5	565	50.0	81.1	14.7	(79.9, 82.3)	85
Age												
< 25	54	9.6	84.4	11.7	(81.2, 87.5)	85.0	53	9.4	85.4	11.9	(82.2, 88.7)	90
25–34	118	20.9	84.6	10.0	(82.8, 86.4)	85.0	130	23.0	84.4	10.6	(82.6, 86.2)	85
35–44	86	15.2	78.1	15.4	(74.8, 81.4)	80.0	95	16.8	78.6	15.0	(75.5, 81.6)	80
45–54	110	19.5	76.2	19.4	(72.6, 79.9)	80.0	102	18.1	75.6	17.7	(72.1, 79.1)	80
55–64	91	16.1	76.5	20.8	(72.2, 80.8)	80.0	67	11.9	82.0	15.5	(78.2, 85.8)	85
65–74	70	12.4	81.7	14.5	(78.3, 85.2)	85.0	57	10.1	79.6	15.8	(75.4, 83.8)	80
75+	35	6.2	76.2	18.1	(70, 82.4)	80.0	61	10.8	84.0	13.3	(80.6, 87.4)	90
General health												
Excellent	118	20.9	91.2	10.2	(89.3, 93.0)	92	109	19.3	92.2	8.2	(90.6, 93.8)	92
Very good	208	36.9	84.6	9.2	(83.4, 85.9)	92	212	37.6	85.3	8.1	(84.2, 86.4)	85
Good	161	28.5	75.8	12.3	(73.9, 77.7)	85	169	30.0	77.2	13.7	(75.1, 79.3)	80
Fair	62	11.0	62.4	19.8	(57.4, 67.4)	80	61	10.8	62.8	16.7	(58.5, 67.0)	60
Poor	15	2.7	36.9	19.7	(26.0, 47.8)	65	13	2.3	56.5	18.6	(45.3, 67.8)	50

The self-reported conditions with the greatest effect on health-related quality of life (HRQoL) included depression, arthritis, and sinusitis with mean utility values of 0.708 (0.258), 0.712 (0.266), and 0.730 (0.279), respectively. (Table 3) The health conditions with the least impact on HRQoL were hypertension: 0.794 (0.223); diabetes mellitus: 0.788 (0.233); and hay fever: 0.785 (0.249).

### Online descriptive statistics and comparison to face-to-face norms

Two thousand and eighteen respondents were recruited from online panels to participate in the online EQ-5D-5L valuation study. Online respondents were also generally representative of the US general population, but the Caucasian race was overrepresented; 77.8% of online respondents were White whereas only 65.5% of the US adult general population was White (Table 1). Similar to the face-to-face sample, online respondents were also more likely to have attained at least secondary education compared to the US general population. Respondents who began but did not complete the survey were not included in these analyses. Two thousand and eighteen respondents completed the online survey, and there was no missing data in this sample.

**Fig. 1:**
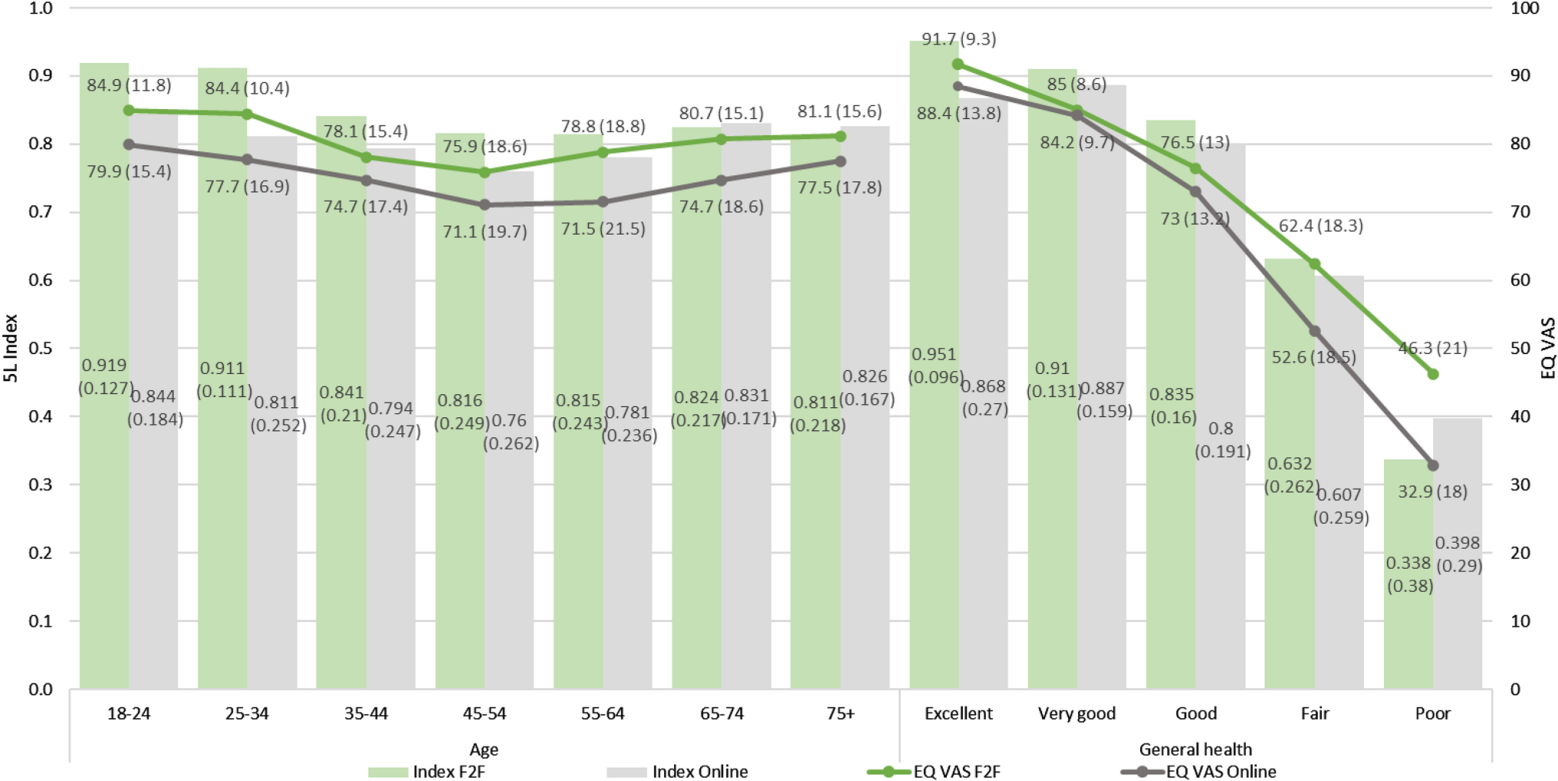
Face-to-face versus Online mean EQ-5D-5L index and EQ VAS by age band and general health. *F2F* Face-to-face, *VAS* visual analogue scale

Of the online sample, 23.9% of the respondents reported no problems on any of the EQ-5D-5L dimensions. (Supplementary material Appendix B) In comparison to face-to-face respondents, online respondents had similar patterns of mean utility and EQ VAS values across different levels of covariates, e.g., decreasing mean norms with worsening general health status and lack of consistent decrease of mean norms with increasing age. (Tables 3–5, Supplementary material Appendices C–E) Online respondents were also more likely to report issues on EQ-5D-5L dimensions with increasing age except for anxiety/depression. (Supplementary material Appendices A, F) Online respondents consistently had lower mean index and EQ VAS values compared to the matching face-to-face age subgroup (Fig. 1). The mean index and EQ VAS values of the overall online sample were 0.800 (0.236) and 74.6 (18.7), respectively, whereas the corresponding values in the face-to-face sample were 0.851 (0.205) and 80.4 (15.6). (Table 3 and Supplementary material Appendix C). For both the EQ VAS and the utility index, the corresponding online values were consistently lower than the face-to-face values across age bands and levels of general health. (Fig. 1).

The effect of mode of administration on index and VAS norms were isolated in linear regressions. After controlling for age, race, gender, ethnicity, and general health status, online respondents had average index and VAS values that were 0.027 and 3.0 units lower than face-to-face respondents, respectively. (Results not shown).

### Comparison of older adult respondents to the US general population

As the plateau effect of norms with increasing age was unexpected, additional comparisons of respondents who were at least 65 years old to the US population were completed. Two hundred and twenty-three face-to-face respondents and 290 online respondents were in this age segment (Supplementary material Appendix G). Face-to-face respondents were more similar to the US general population in terms of distribution of age bands, race, and gender. Both sets of respondents were more educated than the US general population—larger portions of face-to-face and online respondents achieved at least a bachelor’s degree as compared to the US general population. The face-to-face sample was healthier than the respondents recruited for the Medical Expenditure Panel Survey (MEPS), but the comparison of respondent health between the online sample and MEPS respondents was less straightforward.

## Discussion

In this study, we reported age and gender-based norms for the EQ-5D-5L in the US general population. The reference norms were based on data collected from implementation of the EuroQol international standardized protocol using a study design that used quota-based sampling and involved face-to-face, interviewer-assisted administration of the survey and valuation tasks. Data based on the same quotas for sampling collected via online panels provided systematically different respondents and norms, so these were reported separately.

Index-based utility and EQ VAS mean scores did not consistently decrease with increasing age. Instead, the norms were characterized by plateaus or minor increases in older age bands. This pattern may be explained by the lower reported prevalence of anxiety/depression with increasing age as well as different psychometric properties of the EQ-5D-5L (e.g., differential item functioning) and varying priorities for the dimensions by age [35–41]. In the face-to-face sample, approximately 24% of respondents 65 and older reported any problems with anxiety/depression whereas 42.1% of respondents 18 to 24 years old reported the same.(Supplementary material Appendix A) Although issues with mental health may be more often associated with social stigma in older adults and cause fewer older adults to indicate issues with mental health [42], differing levels of social desirability bias due to interviewer presence by age band was unlikely the only contributor to this unexpected distribution by age; the same differential between age bands was observed in the online sample: 24–30% of online respondents 65 and older and 57.1% of respondents 18–24 reported any problems with anxiety/depression (Supplementary material Appendix F).

Similar to the norms reported here, other research corroborates higher prevalence of mental health (e.g., anxiety and depression) issues in US young adults [35–38]. In 2017, the National Institute of Mental Health (NIMH) and the Substance Abuse and Mental Health Services Administration (SAMHSA) found that 4.7% of US adults 50 years and older had a major depressive episode in the past year compared to 13.1% in respondents 18 to 25 years old [35]. Older data from 2001 to 2003 showed that 9.0% of respondents 60 years and older had anxiety disorder in the past year whereas 22.3% of respondents 18 to 29 years old reported the same [36]. Further, mental health reasons for leaving jobs were highest in youngest members of the workforce, and anxiety has also been increasing among young adults since 2008 [37, 38]. In developed, Western countries such as Germany and the UK, some younger segments of the population were also more likely to report mental health issues compared to older segments [43, 44].

Response shift may also have occurred in the older respondents, potentially leading them to interpret or conceptualize the meaning of “no problems” differently from younger respondents who may impose a more ideal expectation when self-reporting their health [39–41, 45, 46]. These interpretation differences could contribute to distinctive psychometric properties of the EQ-5D-5L in different age segments of the population. A combination of increased mental health problems in younger adults and differing interpretation of the problem severity labels could have contributed to the decreased prevalence of anxiety/depression in older respondents and the observed patterns of norms across age groups.

The patterns seen in the US EQ-5D-5L norms are also present in other international norms. Similar to this study, a greater proportion of younger respondents in Chinese urban and Indonesia general populations reported problems with anxiety/depression on the EQ-5D-5L [17, 19]. In China, 34.5% of men 20 to 29 reported any problems with anxiety/depression whereas only 11.5% of men over 70 did the same; the trend was similar in Chinese women [19]. Approximately 40.1% of Indonesian respondents aged 17 to 30 indicated they had any issues with anxiety/depression compared to 32% of respondents older than 50 [17]. Similar to the present study, Canadian, specifically Alberta and Quebec, and Indonesian EQ-5D-5L norms also plateaued in adjacent, older age groups [17, 18, 47].

The systematically lower mean utility and VAS values noted in online respondents relative to face-to-face respondents were likely affected by a combination of factors. Social desirability bias may have contributed to the observed disparities between samples. A greater proportion of online respondents reported issues across all EQ-5D-5L dimensions compared to face-to-face respondents (Supplementary material Appendices D and E). Further, a smaller portion of online respondents indicated that their general health was excellent compared to the face-to-face respondents (12.1% versus 20%; Table 1). If the populations had similar health, face-to-face respondents may have been unwilling to admit health issues in front of an interviewer due to social desirability bias. Past research also found that self-reported health and norms differed when surveys were self-administered versus interviewer-administered via telephone [48].

However, respondents could also have truly differed between modes of data collection due to the varying selection pressures of recruitment and survey needs for each mode of data collection [49]. US online panels tend to be disproportionately White and unrepresentative of minorities [50, 51]. The company which administered the online surveys used in this study noted comparable patterns of minority under-representation in the online panels they employ, which contributed to the low prevalence of non-Black minorities recruited into the online sample [52]. In addition, online respondents must have reliable access to internet and a computer with which to access it and belong to a survey panel to be selected for the study. Face-to-face respondents needed to participate in the study at centralized locations, potentially requiring transportation to attend interviews outside of their homes. Respondent characteristics which determine agreement to survey participation may additionally differ by mode. Distinctions between samples were most noticeable when respondent characteristics unlikely to be susceptible to social desirability bias were examined. For example, online respondents were more likely to have children under 18 than face-to-face respondents (Table 1). Finally, face-to-face respondents aged 65 and older were more comparable to the US general population than these respondents in the online sample, particularly in terms of age and gender distribution. (Supplementary material Appendix G).

As the online population appeared to be less representative of the US general population, the two samples were kept distinct. Based on the aforementioned differences between online and face-to-face respondents as well as the improved representativeness in older respondents, the face-to-face norms should be the primary set of normative values for the US population, particularly for data collected in-person. The online norms may be most useful for comparison of unsupervised data elicited from online panels.

The study had several limitations. Quota-sampling was employed for practical time and cost considerations, and random sampling may have allowed for a more representative sample of the US population, particularly in the face-to-face sample. This shortcoming can be observed in the education attainment and general health of respondents who were at least 65 years old (Supplementary material Appendix G). The sample sizes included in the present study were relatively small in comparison to the US population size, and each face-to-face respondent represented approximately 290,000 US inhabitants. As the EQ-5D-5L is not included in any large-scale US general population surveys, these data were the best available sources to estimate US general population norms. Finally, the mean index and VAS values estimated for each diagnosis in both online and face-to-face samples should only be used as a general guidance for disease burden as these subgroups are likely heterogenous in disease stage, acuity, and patient experience.

With the present study, US EQ-5D-5L utility and EQ VAS norms are now available as general population benchmarks to support health services research across research, clinical, and policy settings for two major methods of data collection. End-users of the EQ-5D-5L may choose the set most appropriate for the application.

## Electronic supplementary material

Below is the link to the electronic supplementary material.

Supplementary file1 (DOCX 124 kb)

## Data Availability

Data available upon request were submitted to the corresponding author.
