# Supplementary material for: US population norms for the EQ-5D-5L and comparison of norms from face-to-face and online samples
Source: Qual Life Res. 2020 Oct 6;30(3):803–16. doi: 10.1007/s11136-020-02650-y (PMC7952367; doi:10.1007/s11136-020-02650-y)
Supplement: Supplementary file 1 — Supplementary file1 (DOCX 124 kb) [file 11136_2020_2650_MOESM1_ESM.docx]

Appendix A US Face-to-face sample frequency of self-reported problems by dimension-level and gender

|  |  | **Face-to-face Online sample** | | | | | | | | | | | | | | **Face-to-face male sample** | | | | | | | | | | | | | | **Face-to-face female sample** | | | | | | | | | | | | | |
| --- | --- | --- | --- | --- | --- | --- | --- | --- | --- | --- | --- | --- | --- | --- | --- | --- | --- | --- | --- | --- | --- | --- | --- | --- | --- | --- | --- | --- | --- | --- | --- | --- | --- | --- | --- | --- | --- | --- | --- | --- | --- | --- | --- |
| **Age band** | | **<25** | | **25-34** | | **35-44** | | **45-54** | | **55-64** | | **65-74** | | **75+** | | **<25** | | **25-34** | | **35-44** | | **45-54** | | **55-64** | | **65-74** | | **75+** | | **<25** | | **25-34** | | **35-44** | | **45-54** | | **55-64** | | **65-74** | | **75+** | |
| **MO** | **1** | 102 | 95.3% | 225 | 89.6% | 139 | 76.4% | 140 | 66.0% | 96 | 60.4% | 71 | 55.9% | 39 | 40.6% | 51 | 94.4% | 105 | 89.0% | 64 | 74.4% | 79 | 71.8% | 56 | 61.5% | 36 | 51.4% | 13 | 37.1% | 51 | 96.2% | 118 | 90.8% | 74 | 77.9% | 61 | 59.8% | 40 | 59.7% | 35 | 61.4% | 26 | 42.6% |
|  | **2** | 4 | 3.7% | 21 | 8.4% | 29 | 15.9% | 44 | 20.8% | 44 | 27.7% | 41 | 32.3% | 25 | 26.0% | 2 | 3.7% | 10 | 8.5% | 13 | 15.1% | 16 | 14.5% | 25 | 27.5% | 26 | 37.1% | 7 | 20.0% | 2 | 3.8% | 10 | 7.7% | 16 | 16.8% | 28 | 27.5% | 18 | 26.9% | 15 | 26.3% | 18 | 29.5% |
|  | **3** | 1 | 0.9% | 5 | 2.0% | 11 | 6.0% | 16 | 7.5% | 10 | 6.3% | 9 | 7.1% | 27 | 28.1% | 1 | 1.9% | 3 | 2.5% | 7 | 8.1% | 6 | 5.5% | 3 | 3.3% | 4 | 5.7% | 13 | 37.1% | 0 | 0.0% | 2 | 1.5% | 4 | 4.2% | 10 | 9.8% | 7 | 10.4% | 5 | 8.8% | 14 | 23.0% |
|  | **4** | 0 | 0.0% | 0 | 0.0% | 3 | 1.6% | 11 | 5.2% | 9 | 5.7% | 4 | 3.1% | 4 | 4.2% | 0 | 0.0% | 0 | 0.0% | 2 | 2.3% | 8 | 7.3% | 7 | 7.7% | 2 | 2.9% | 1 | 2.9% | 0 | 0.0% | 0 | 0.0% | 1 | 1.1% | 3 | 2.9% | 2 | 3.0% | 2 | 3.5% | 3 | 4.9% |
|  | **5** | 0 | 0.0% | 0 | 0.0% | 0 | 0.0% | 1 | 0.5% | 0 | 0.0% | 2 | 1.6% | 1 | 1.0% | 0 | 0.0% | 0 | 0.0% | 0 | 0.0% | 1 | 0.9% | 0 | 0.0% | 2 | 2.9% | 1 | 2.9% | 0 | 0.0% | 0 | 0.0% | 0 | 0.0% | 0 | 0.0% | 0 | 0.0% | 0 | 0.0% | 0 | 0.0% |
| **SC** | **1** | 106 | 99.1% | 241 | 96.0% | 174 | 95.6% | 194 | 91.5% | 141 | 88.7% | 119 | 93.7% | 85 | 88.5% | 53 | 98.1% | 112 | 94.9% | 81 | 94.2% | 103 | 93.6% | 79 | 86.8% | 66 | 94.3% | 31 | 88.6% | 53 | ##### | 126 | 96.9% | 92 | 96.8% | 91 | 89.2% | 61 | 91.0% | 53 | 93.0% | 54 | 88.5% |
|  | **2** | 1 | 0.9% | 9 | 3.6% | 4 | 2.2% | 11 | 5.2% | 10 | 6.3% | 4 | 3.1% | 3 | 3.1% | 1 | 1.9% | 5 | 4.2% | 2 | 2.3% | 3 | 2.7% | 5 | 5.5% | 2 | 2.9% | 0 | 0.0% | 0 | 0.0% | 4 | 3.1% | 2 | 2.1% | 8 | 7.8% | 5 | 7.5% | 2 | 3.5% | 3 | 4.9% |
|  | **3** | 0 | 0.0% | 0 | 0.0% | 2 | 1.1% | 6 | 2.8% | 8 | 5.0% | 2 | 1.6% | 7 | 7.3% | 0 | 0.0% | 0 | 0.0% | 2 | 2.3% | 3 | 2.7% | 7 | 7.7% | 1 | 1.4% | 3 | 8.6% | 0 | 0.0% | 0 | 0.0% | 0 | 0.0% | 3 | 2.9% | 1 | 1.5% | 1 | 1.8% | 4 | 6.6% |
|  | **4** | 0 | 0.0% | 0 | 0.0% | 2 | 1.1% | 1 | 0.5% | 0 | 0.0% | 2 | 1.6% | 0 | 0.0% | 0 | 0.0% | 0 | 0.0% | 1 | 1.2% | 1 | 0.9% | 0 | 0.0% | 1 | 1.4% | 0 | 0.0% | 0 | 0.0% | 0 | 0.0% | 1 | 1.1% | 0 | 0.0% | 0 | 0.0% | 1 | 1.8% | 0 | 0.0% |
|  | **5** | 0 | 0.0% | 1 | 0.4% | 0 | 0.0% | 0 | 0.0% | 0 | 0.0% | 0 | 0.0% | 1 | 1.0% | 0 | 0.0% | 1 | 0.8% | 0 | 0.0% | 0 | 0.0% | 0 | 0.0% | 0 | 0.0% | 1 | 2.9% | 0 | 0.0% | 0 | 0.0% | 0 | 0.0% | 0 | 0.0% | 0 | 0.0% | 0 | 0.0% | 0 | 0.0% |
| **UA** | **1** | 96 | 89.7% | 216 | 86.1% | 129 | 70.9% | 154 | 72.6% | 110 | 69.2% | 85 | 66.9% | 64 | 66.7% | 50 | 92.6% | 100 | 84.7% | 68 | 79.1% | 81 | 73.6% | 63 | 69.2% | 52 | 74.3% | 22 | 62.9% | 46 | 86.8% | 114 | 87.7% | 61 | 64.2% | 73 | 71.6% | 47 | 70.1% | 33 | 57.9% | 42 | 68.9% |
|  | **2** | 9 | 8.4% | 28 | 11.2% | 38 | 20.9% | 24 | 11.3% | 32 | 20.1% | 28 | 22.0% | 19 | 19.8% | 3 | 5.6% | 14 | 11.9% | 11 | 12.8% | 13 | 11.8% | 16 | 17.6% | 11 | 15.7% | 9 | 25.7% | 6 | 11.3% | 13 | 10.0% | 26 | 27.4% | 11 | 10.8% | 15 | 22.4% | 17 | 29.8% | 10 | 16.4% |
|  | **3** | 1 | 0.9% | 6 | 2.4% | 11 | 6.0% | 27 | 12.7% | 13 | 8.2% | 12 | 9.4% | 10 | 10.4% | 0 | 0.0% | 4 | 3.4% | 5 | 5.8% | 13 | 11.8% | 9 | 9.9% | 6 | 8.6% | 3 | 8.6% | 1 | 1.9% | 2 | 1.5% | 6 | 6.3% | 14 | 13.7% | 4 | 6.0% | 6 | 10.5% | 7 | 11.5% |
|  | **4** | 0 | 0.0% | 1 | 0.4% | 3 | 1.6% | 6 | 2.8% | 3 | 1.9% | 2 | 1.6% | 1 | 1.0% | 0 | 0.0% | 0 | 0.0% | 2 | 2.3% | 3 | 2.7% | 2 | 2.2% | 1 | 1.4% | 0 | 0.0% | 0 | 0.0% | 1 | 0.8% | 1 | 1.1% | 3 | 2.9% | 1 | 1.5% | 1 | 1.8% | 1 | 1.6% |
|  | **5** | 1 | 0.9% | 0 | 0.0% | 1 | 0.5% | 1 | 0.5% | 1 | 0.6% | 0 | 0.0% | 2 | 2.1% | 1 | 0.0% | 0 | 0.0% | 0 | 0.0% | 0 | 0.0% | 1 | 1.1% | 0 | 0.0% | 1 | 2.9% | 0 | 0.0% | 0 | 0.0% | 1 | 1.1% | 1 | 1.0% | 0 | 0.0% | 0 | 0.0% | 1 | 1.6% |
| **PD** | **1** | 72 | 67.3% | 150 | 59.8% | 83 | 45.6% | 96 | 45.3% | 68 | 42.8% | 46 | 36.2% | 41 | 42.7% | 35 | 64.8% | 66 | 55.9% | 39 | 45.3% | 53 | 48.2% | 41 | 45.1% | 24 | 34.3% | 14 | 40.0% | 37 | 69.8% | 83 | 63.8% | 44 | 46.3% | 43 | 42.2% | 27 | 40.3% | 22 | 38.6% | 27 | 44.3% |
|  | **2** | 29 | 27.1% | 77 | 30.7% | 63 | 34.6% | 64 | 30.2% | 51 | 32.1% | 55 | 43.3% | 35 | 36.5% | 14 | 25.9% | 37 | 31.4% | 30 | 34.9% | 31 | 28.2% | 30 | 33.0% | 33 | 47.1% | 13 | 37.1% | 15 | 28.3% | 38 | 29.2% | 33 | 34.7% | 33 | 32.4% | 20 | 29.9% | 22 | 38.6% | 22 | 36.1% |
|  | **3** | 5 | 4.7% | 22 | 8.8% | 27 | 14.8% | 33 | 15.6% | 28 | 17.6% | 19 | 15.0% | 17 | 17.7% | 4 | 7.4% | 14 | 11.9% | 12 | 14.0% | 16 | 14.5% | 12 | 13.2% | 8 | 11.4% | 6 | 17.1% | 1 | 1.9% | 8 | 6.2% | 14 | 14.7% | 17 | 16.7% | 16 | 23.9% | 11 | 19.3% | 11 | 18.0% |
|  | **4** | 1 | 0.9% | 2 | 0.8% | 7 | 3.8% | 16 | 7.5% | 6 | 3.8% | 5 | 3.9% | 2 | 2.1% | 1 | 1.9% | 1 | 0.8% | 4 | 4.7% | 8 | 7.3% | 5 | 5.5% | 4 | 5.7% | 1 | 2.9% | 0 | 0.0% | 1 | 0.8% | 3 | 3.2% | 8 | 7.8% | 1 | 1.5% | 1 | 1.8% | 1 | 1.6% |
|  | **5** | 0 | 0.0% | 0 | 0.0% | 2 | 1.1% | 3 | 1.4% | 6 | 3.8% | 2 | 1.6% | 1 | 1.0% | 0 | 0.0% | 0 | 0.0% | 1 | 1.2% | 2 | 1.8% | 3 | 3.3% | 1 | 1.4% | 1 | 2.9% | 0 | 0.0% | 0 | 0.0% | 1 | 1.1% | 1 | 1.0% | 3 | 4.5% | 1 | 1.8% | 0 | 0.0% |
| **AD** | **1** | 62 | 57.9% | 143 | 57.0% | 95 | 52.2% | 131 | 61.8% | 98 | 61.6% | 97 | 76.4% | 73 | 76.0% | 33 | 61.1% | 69 | 58.5% | 40 | 46.5% | 74 | 67.3% | 55 | 60.4% | 60 | 85.7% | 26 | 74.3% | 29 | 54.7% | 74 | 56.9% | 55 | 57.9% | 57 | 55.9% | 43 | 64.2% | 37 | 64.9% | 47 | 77.0% |
|  | **2** | 28 | 26.2% | 80 | 31.9% | 50 | 27.5% | 47 | 22.2% | 38 | 23.9% | 14 | 11.0% | 15 | 15.6% | 10 | 18.5% | 39 | 33.1% | 29 | 33.7% | 20 | 18.2% | 22 | 24.2% | 5 | 7.1% | 7 | 20.0% | 18 | 34.0% | 39 | 30.0% | 21 | 22.1% | 27 | 26.5% | 15 | 22.4% | 9 | 15.8% | 8 | 13.1% |
|  | **3** | 12 | 11.2% | 25 | 10.0% | 29 | 15.9% | 25 | 11.8% | 21 | 13.2% | 12 | 9.4% | 7 | 7.3% | 7 | 13.0% | 9 | 7.6% | 15 | 17.4% | 10 | 9.1% | 12 | 13.2% | 2 | 2.9% | 1 | 2.9% | 5 | 9.4% | 15 | 11.5% | 14 | 14.7% | 15 | 14.7% | 9 | 13.4% | 10 | 17.5% | 6 | 9.8% |
|  | **4** | 3 | 2.8% | 3 | 1.2% | 7 | 3.8% | 6 | 2.8% | 1 | 0.6% | 3 | 2.4% | 1 | 1.0% | 2 | 3.7% | 1 | 0.8% | 2 | 2.3% | 4 | 3.6% | 1 | 1.1% | 2 | 2.9% | 1 | 2.9% | 1 | 1.9% | 2 | 1.5% | 4 | 4.2% | 2 | 2.0% | 0 | 0.0% | 1 | 1.8% | 0 | 0.0% |
|  | **5** | 2 | 1.9% | 0 | 0.0% | 1 | 0.5% | 3 | 1.4% | 1 | 0.6% | 1 | 0.8% | 0 | 0.0% | 2 | 3.7% | 0 | 0.0% | 0 | 0.0% | 2 | 1.8% | 1 | 1.1% | 1 | 1.4% | 0 | 0.0% | 0 | 0.0% | 0 | 0.0% | 1 | 1.1% | 1 | 1.0% | 0 | 0.0% | 0 | 0.0% | 0 | 0.0% |

Appendix B Most frequently reported EQ-5D-5L health states in the Online sample (prevalence ≥ 0.5%)

| EQ-5D-5L  health state | N | % | EQ-5D-5L  health state | N | % |
| --- | --- | --- | --- | --- | --- |
| 11111 | 478 | 23.7% | 11133 | 20 | 1% |
| 11121 | 213 | 10.6% | 11222 | 18 | 0.9% |
| 11122 | 167 | 8.3% | 21122 | 18 | 0.9% |
| 11112 | 120 | 6% | 21222 | 18 | 0.9% |
| 11123 | 76 | 3.8% | 11223 | 17 | 0.8% |
| 11113 | 71 | 3.5% | 11114 | 16 | 0.8% |
| 11131 | 33 | 1.6% | 21233 | 15 | 0.7% |
| 21121 | 31 | 1.5% | 33333 | 15 | 0.7% |
| 21221 | 31 | 1.5% | 22222 | 13 | 0.6% |
| 11221 | 29 | 1.4% | 11124 | 11 | 0.6% |
| 11132 | 25 | 1.2% | 11231 | 11 | 0.6% |
| 21232 | 25 | 1.2% | 21223 | 11 | 0.6% |
| 21231 | 21 | 1% | 21132 | 10 | 0.5% |

Appendix C Online index and VAS-based norms by respondent characteristics

|  | **US EQ-5D-5L Index** | | | | | | | **EQ VAS** | | | | | |
| --- | --- | --- | --- | --- | --- | --- | --- | --- | --- | --- | --- | --- | --- |
|  | **n** | **%** | **Mean** | **Standard**  **Deviation** | **95% CI** | **Median** | **P-value** | **Mean** | **Standard**  **Deviation** | **95% CI** | **Median** | **P-value** |  |
| **Overall** | **2018** | 100% | 0.800 | 0.236 | (0.789, 0.809) | 0.883 |  | 74.6 | 18.7 | (73.8, 75.5) | 80 |  |  |
| **Age** |  |  |  |  |  |  |  |  |  |  |  |  |  |
| **<25** | 133 | 6.6% | 0.844 | 0.184 | (0.812, 0.876) | 0.883 | <0.001 | 79.9 | 15.4 | (77.3, 82.6) | 83 | <0.001 |  |
| **25-34** | 494 | 24.5% | 0.811 | 0.252 | (0.789, 0.834) | 0.883 |  | 77.7 | 16.9 | (76.2, 79.2) | 81 |  |  |
| **35-44** | 385 | 19.1% | 0.794 | 0.247 | (0.77, 0.819) | 0.883 |  | 74.7 | 17.4 | (73, 76.4) | 80 |  |  |
| **45-54** | 330 | 16.4% | 0.760 | 0.262 | (0.732, 0.789) | 0.845 |  | 71.1 | 19.7 | (69, 73.3) | 78 |  |  |
| **55-64** | 386 | 19.1% | 0.781 | 0.236 | (0.758, 0.805) | 0.845 |  | 71.5 | 21.5 | (69.4, 73.7) | 79 |  |  |
| **65-74** | 252 | 12.5% | 0.831 | 0.171 | (0.81, 0.853) | 0.877 |  | 74.7 | 18.6 | (72.4, 77) | 80 |  |  |
| **75+** | 38 | 1.9% | 0.826 | 0.167 | (0.771, 0.88) | 0.860 |  | 77.5 | 17.8 | (71.7, 83.4) | 80 |  |  |
| **Gender** |  |  |  |  |  |  |  |  |  |  |  |  |  |
| **Male** | 973 | 48.3% | 0.792 | 0.256 | (0.776, 0.808) | 0.883 | 0.180 | 74.5 | 19.1 | (73.3, 75.7) | 80 | 0.618 |  |
| **Female** | 1041 | 51.7% | 0.806 | 0.216 | (0.793, 0.819) | 0.883 |  | 74.9 | 18.3 | (73.8, 76) | 80 |  |  |
| **Race category** |  |  |  |  |  |  |  |  |  |  |  |  |  |
| **White** | 1570 | 77.8% | 0.795 | 0.232 | (0.784, 0.807) | 0.877 | 0.147 | 73.9 | 19.0 | (72.9, 74.8) | 80 | 0.001 |  |
| **Black** | 258 | 12.8% | 0.798 | 0.266 | (0.765, 0.830) | 0.902 |  | 78.1 | 18.3 | (75.9, 80.3) | 82 |  |  |
| **Other** | 190 | 9.4% | 0.831 | 0.229 | (0.798, 0.864) | 0.883 |  | 76.4 | 16.3 | (74.1 ,78.7) | 80 |  |  |
| **Ethnicity** |  |  |  |  |  |  |  |  |  |  |  |  |  |
| **Hispanic** | 308 | 15.3% | 0.760 | 0.268 | (0.730, 0.790) | 0.863 | 0.002 | 75.7 | 18.1 | (73.7, 77.8) | 80 | 0.268 |  |
| **Not Hispanic** | 1710 | 84.7% | 0.806 | 0.230 | (0.795, 0.817) | 0.883 |  | 74.5 | 18.8 | (73.6, 75.3) | 80 |  |  |
| **General Health** |  |  |  |  |  |  |  |  |  |  |  |  |  |
| **Excellent** | 245 | 12.1% | 0.868 | 0.270 | (0.834, 0.902) | 1 | <0.001 | 88.4 | 13.8 | (86.7, 90.1) | 91 | <0.001 |  |
| **Very good** | 695 | 34.4% | 0.887 | 0.159 | (0.875, 0.899) | 0.94 |  | 84.2 | 9.7 | (83.4, 84.9) | 85 |  |  |
| **Good** | 730 | 36.2% | 0.800 | 0.191 | (0.786, 0.814) | 0.845 |  | 73.0 | 13.2 | (72.1, 74) | 75 |  |  |
| **Fair** | 290 | 14.4% | 0.607 | 0.259 | (0.577, 0.637) | 0.648 |  | 52.6 | 18.5 | (50.5, 54.8) | 54.5 |  |  |
| **Poor** | 58 | 2.9% | 0.398 | 0.290 | (0.322, 0.475) | 0.402 |  | 32.9 | 18.0 | (28.1, 37.6) | 30 |  |  |
| **Regular prescription medications** |  |  |  |  |  |  |  |  |  |  |  |  |  |
| **0** | 819 | 40.6% | 0.887 | 0.156 | (0.876, 0.897) | 0.94 | <0.001 | 80.0 | 15.1 | (78.9, 81) | 82 | <0.001 |  |
| **1** | 393 | 19.5% | 0.820 | 0.227 | (0.797, 0.842) | 0.883 |  | 78.2 | 15.3 | (76.6, 79.7) | 80 |  |  |
| **2 to 4** | 559 | 27.7% | 0.735 | 0.252 | (0.714, 0.756) | 0.815 |  | 71.2 | 19.7 | (69.5, 72.8) | 76 |  |  |
| **5 or more** | 247 | 12.2% | 0.618 | 0.288 | (0.582, 0.654) | 0.678 |  | 59.3 | 22.2 | (56.5, 62.1) | 62 |  |  |
| **Personal experience with serious illness** |  |  |  |  |  |  |  |  |  |  |  |  |  |
| **No** | 1380 | 68.4% | 0.870 | 0.158 | (0.862, 0.878) | 0.94 | <0.001 | 79.5 | 15.0 | (78.8, 80.3) | 82 | <0.001 |  |
| **Yes** | 638 | 31.6% | 0.645 | 0.297 | (0.622, 0.668) | 0.701 |  | 64.0 | 21.4 | (62.4, 65.7) | 70 |  |  |
| **Family experience with serious illness** |  |  |  |  |  |  |  |  |  |  |  |  |  |
| **No** | 712 | 35.3% | 0.853 | 0.203 | (0.838, 0.868) | 0.94 | <0.001 | 78.0 | 15.0 | (76.7, 79.3) | 81 | 0.054 |  |
| **Yes** | 1306 | 64.7% | 0.770 | 0.248 | (0.756, 0.783) | 0.845 |  | 72.8 | 21.4 | (71.8, 73.8) | 79 |  |  |
| **Experience caring for someone with serious illness** |  |  |  |  |  |  |  |  |  |  |  |  |  |
| **No** | 1132 | 56.1% | 0.828 | 0.222 | (0.815, 0.841) | 0.883 | <0.001 | 75.8 | 18.2 | (74.8, 76.9) | 80 | 0.007 |  |
| **Yes** | 886 | 43.9% | 0.762 | 0.249 | (0.745, 0.778) | 0.844 |  | 73.1 | 19.3 | (71.8, 74.4) | 80 |  |  |
| **Health condition** |  |  |  |  |  |  |  |  |  |  |  |  |  |
| **Arthritis** | 445 | 22.1% | 0.655 | 0.285 | (0.629, 0.682) | 0.712 |  | 65.8 | 21.1 | (63.8, 67.7) | 70 |  |  |
| **Asthma** | 195 | 9.7% | 0.717 | 0.258 | (0.681, 0.754) | 0.779 |  | 67.8 | 21.2 | (64.8, 70.8) | 74 |  |  |
| **Depression** | 438 | 21.7% | 0.645 | 0.251 | (0.621, 0.668) | 0.701 |  | 64.7 | 21.2 | (62.7, 66.7) | 70 |  |  |
| **DM** | 223 | 11.1% | 0.689 | 0.300 | (0.650, 0.729) | 0.776 |  | 67.4 | 19.9 | (64.8, 70.0) | 71 |  |  |
| **Hay fever** | 199 | 9.9% | 0.784 | 0.225 | (0.753, 0.816) | 0.872 |  | 70.2 | 20.5 | (67.3, 73.1) | 75 |  |  |
| **Hypertension** | 507 | 25.1% | 0.734 | 0.260 | (0.711, 0.757) | 0.806 |  | 69.2 | 20.1 | (67.5, 71.0) | 75 |  |  |
| **Migraine** | 232 | 11.5% | 0.688 | 0.265 | (0.654, 0.722) | 0.749 |  | 68.0 | 20.8 | (65.3, 70.7) | 72 |  |  |
| **Sinusitis** | 171 | 8.5% | 0.710 | 0.264 | (0.670, 0.750) | 0.777 |  | 67.6 | 21.2 | (64.4, 70.8) | 75 |  |  |

Appendix D Online index and VAS-based norms by EQ-5D-5L dimension-levels and gender

|  |  |  | **Total** | | | | | | **Men** | | | | | | **Women** | | | | | | | |
| --- | --- | --- | --- | --- | --- | --- | --- | --- | --- | --- | --- | --- | --- | --- | --- | --- | --- | --- | --- | --- | --- | --- |
|  |  |  | **n** | **%** | **Mean** | **SD** | **95% CI** | **Median** | **n** | **%** | **Mean** | **SD** | **95% CI** | **Median** | | **n** | **%** | **Mean** | **SD** | **95% CI** | **Median** |  |
| **EQ-5D-5L index** | **Mobility** | **1** | 1425 | 70.6% | 0.903 | 0.120 | (0.897, 0.909) | 0.94 | 661 | 67.9% | 0.915 | 0.116 | (0.906, 0.924) | 0.940 | | 761 | 73.1% | 0.894 | 0.122 | (0.885, 0.902) | 0.940 |  |
|  |  | **2** | 361 | 17.9% | 0.651 | 0.170 | (0.633, 0.668) | 0.683 | 176 | 18.1% | 0.652 | 0.172 | (0.627, 0.678) | 0.687 | | 184 | 17.7% | 0.650 | 0.168 | (0.626, 0.675) | 0.681 |  |
|  |  | **3** | 172 | 8.5% | 0.468 | 0.202 | (0.437, 0.498) | 0.488 | 100 | 10.3% | 0.461 | 0.202 | (0.421, 0.501) | 0.485 | | 72 | 6.9% | 0.477 | 0.203 | (0.429, 0.525) | 0.498 |  |
|  |  | **4** | 42 | 2.1% | 0.153 | 0.321 | (0.053, 0.253) | 0.146 | 24 | 2.5% | 0.177 | 0.314 | (0.045, 0.31) | 0.173 | | 18 | 1.7% | 0.121 | 0.336 | (-0.046, 0.288) | 0.060 |  |
|  |  | **5** | 18 | 0.9% | 0.197 | 0.505 | (-0.054, 0.448) | 0.3525 | 12 | 1.2% | 0.051 | 0.559 | (-0.304, 0.406) | 0.209 | | 6 | 0.6% | 0.488 | 0.159 | (0.321, 0.655) | 0.461 |  |
|  | **Self-care** | **1** | 1755 | 87.0% | 0.858 | 0.158 | (0.851, 0.865) | 0.883 | 803 | 82.5% | 0.872 | 0.155 | (0.862, 0.883) | 0.940 | | 949 | 91.2% | 0.847 | 0.159 | (0.836, 0.857) | 0.883 |  |
|  |  | **2** | 173 | 8.6% | 0.508 | 0.209 | (0.477, 0.539) | 0.566 | 102 | 10.5% | 0.534 | 0.202 | (0.495, 0.574) | 0.591 | | 70 | 6.7% | 0.469 | 0.217 | (0.418, 0.521) | 0.530 |  |
|  |  | **3** | 65 | 3.2% | 0.309 | 0.225 | (0.253, 0.364) | 0.37 | 48 | 4.9% | 0.352 | 0.186 | (0.298, 0.406) | 0.444 | | 17 | 1.6% | 0.185 | 0.280 | (0.041, 0.329) | 0.229 |  |
|  |  | **4** | 15 | 0.7% | 0.020 | 0.331 | (-0.163, 0.203) | 0.006 | 12 | 1.2% | 0.048 | 0.319 | (-0.154, 0.251) | 0.059 | | 3 | 0.3% | -0.093 | 0.429 | (-1.157, 0.972) | -0.329 |  |
|  |  | **5** | 10 | 0.5% | -0.182 | 0.436 | (-0.494, 0.131) | -0.364 | 8 | 0.8% | -0.235 | 0.453 | (-0.614, 0.143) | -0.525 | | 2 | 0.2% | 0.034 | 0.403 | (-3.587, 3.655) | 0.034 |  |
|  | **Usual activities** | **1** | 1389 | 68.8% | 0.909 | 0.104 | (0.904, 0.915) | 0.94 | 647 | 66.5% | 0.919 | 0.104 | (0.911, 0.927) | 0.940 | | 741 | 71.2% | 0.901 | 0.103 | (0.894, 0.909) | 0.940 |  |
|  |  | **2** | 385 | 19.1% | 0.675 | 0.150 | (0.660, 0.690) | 0.69 | 195 | 20.0% | 0.676 | 0.162 | (0.653, 0.699) | 0.711 | | 189 | 18.2% | 0.676 | 0.136 | (0.656, 0.695) | 0.681 |  |
|  |  | **3** | 187 | 9.3% | 0.473 | 0.184 | (0.447, 0.500) | 0.485 | 97 | 10.0% | 0.460 | 0.186 | (0.422, 0.497) | 0.475 | | 88 | 8.5% | 0.485 | 0.182 | (0.447, 0.524) | 0.498 |  |
|  |  | **4** | 46 | 2.3% | 0.063 | 0.239 | (-0.008, 0.134) | 0.075 | 26 | 2.7% | 0.080 | 0.212 | (-0.005, 0.166) | 0.107 | | 20 | 1.9% | 0.041 | 0.275 | (-0.088, 0.17) | 0.016 |  |
|  |  | **5** | 11 | 0.5% | -0.224 | 0.356 | (-0.463, 0.015) | -0.251 | 8 | 0.8% | -0.303 | 0.361 | (-0.605, -0.002) | -0.525 | | 3 | 0.3% | -0.012 | 0.292 | (-0.736, 0.713) | -0.097 |  |
|  | **Pain/**  **discomfort** | **1** | 749 | 37.1% | 0.950 | 0.097 | (0.943, 0.957) | 1 | 392 | 40.3% | 0.952 | 0.105 | (0.941, 0.962) | 1.000 | | 356 | 34.2% | 0.950 | 0.085 | (0.941, 0.958) | 1.000 |  |
|  |  | **2** | 760 | 37.7% | 0.826 | 0.125 | (0.817, 0.834) | 0.8775 | 331 | 34.0% | 0.821 | 0.135 | (0.807, 0.836) | 0.872 | | 426 | 40.9% | 0.830 | 0.116 | (0.819, 0.841) | 0.883 |  |
|  |  | **3** | 383 | 19.0% | 0.648 | 0.175 | (0.631, 0.666) | 0.681 | 190 | 19.5% | 0.617 | 0.183 | (0.591, 0.644) | 0.649 | | 193 | 18.5% | 0.679 | 0.160 | (0.656, 0.701) | 0.681 |  |
|  |  | **4** | 104 | 5.2% | 0.257 | 0.233 | (0.212, 0.302) | 0.282 | 49 | 5.0% | 0.219 | 0.227 | (0.154, 0.284) | 0.247 | | 55 | 5.3% | 0.291 | 0.235 | (0.228, 0.355) | 0.339 |  |
|  |  | **5** | 22 | 1.1% | -0.084 | 0.342 | (-0.235, 0.068) | -0.091 | 11 | 1.1% | -0.209 | 0.390 | (-0.471, 0.053) | -0.136 | | 11 | 1.1% | 0.042 | 0.242 | (-0.121, 0.204) | 0.037 |  |
|  | **Anxiety/**  **depression** | **1** | 987 | 48.9% | 0.911 | 0.141 | (0.902, 0.920) | 0.94 | 518 | 53.2% | 0.913 | 0.139 | (0.901, 0.925) | 1.000 | | 469 | 45.1% | 0.910 | 0.142 | (0.897, 0.923) | 0.940 |  |
|  |  | **2** | 524 | 26.0% | 0.801 | 0.161 | (0.787, 0.815) | 0.883 | 232 | 23.8% | 0.794 | 0.166 | (0.773, 0.816) | 0.883 | | 292 | 28.1% | 0.807 | 0.158 | (0.788, 0.825) | 0.883 |  |
|  |  | **3** | 349 | 17.3% | 0.673 | 0.217 | (0.650, 0.696) | 0.749 | 148 | 15.2% | 0.612 | 0.246 | (0.573, 0.652) | 0.683 | | 199 | 19.1% | 0.717 | 0.181 | (0.692, 0.743) | 0.817 |  |
|  |  | **4** | 100 | 5.0% | 0.439 | 0.244 | (0.391, 0.488) | 0.507 | 44 | 4.5% | 0.346 | 0.251 | (0.27, 0.422) | 0.350 | | 55 | 5.3% | 0.512 | 0.216 | (0.454, 0.571) | 0.573 |  |
|  |  | **5** | 58 | 2.9% | 0.244 | 0.388 | (0.142, 0.347) | 0.417 | 31 | 3.2% | 0.248 | 0.436 | (0.088, 0.408) | 0.455 | | 26 | 2.5% | 0.232 | 0.338 | (0.096, 0.368) | 0.236 |  |
| **EQ VAS** | **Mobility** | **1** | 1425 | 70.6% | 80.0 | 14.5 | (79.3, 80.8) | 82 | 661 | 67.9% | 80.0 | 15.4 | (78.8, 81.2) | 83.0 | | 761 | 73.1% | 80.1 | 13.5 | (79.2, 81.1) | 81.0 |  |
|  |  | **2** | 361 | 17.9% | 65.0 | 19.8 | (63, 67.1) | 70 | 176 | 18.1% | 65.0 | 19.8 | (62, 67.9) | 69.5 | | 184 | 17.7% | 65.2 | 19.7 | (62.4, 68.1) | 70.0 |  |
|  |  | **3** | 172 | 8.5% | 57.8 | 21.5 | (54.6, 61.1) | 62 | 100 | 10.3% | 61.3 | 20.0 | (57.4, 65.3) | 66.5 | | 72 | 6.9% | 53.0 | 22.8 | (47.6, 58.4) | 53.0 |  |
|  |  | **4** | 42 | 2.1% | 46.1 | 24.6 | (38.4, 53.8) | 40 | 24 | 2.5% | 48.5 | 26.3 | (37.3, 59.6) | 40.5 | | 18 | 1.7% | 42.9 | 22.5 | (31.8, 54.1) | 34.0 |  |
|  |  | **5** | 18 | 0.9% | 70.0 | 19.0 | (60.6, 79.4) | 78 | 12 | 1.2% | 72.2 | 21.6 | (58.4, 85.9) | 81.0 | | 6 | 0.6% | 65.7 | 12.9 | (52.1, 79.2) | 67.5 |  |
|  | **Self-care** | **1** | 1755 | 87.0% | 77.3 | 16.6 | (76.5, 78) | 80 | 803 | 82.5% | 77.4 | 17.1 | (76.2, 78.6) | 80.0 | | 949 | 91.2% | 77.2 | 16.1 | (76.2, 78.3) | 80.0 |  |
|  |  | **2** | 173 | 8.6% | 56.5 | 20.5 | (53.5, 59.6) | 60 | 102 | 10.5% | 59.7 | 20.9 | (55.6, 63.8) | 63.5 | | 70 | 6.7% | 52.2 | 19.4 | (47.5, 56.8) | 53.0 |  |
|  |  | **3** | 65 | 3.2% | 57.4 | 23.8 | (51.5, 63.3) | 60 | 48 | 4.9% | 62.4 | 22.0 | (56, 68.8) | 66.0 | | 17 | 1.6% | 43.4 | 23.8 | (31.2, 55.7) | 35.0 |  |
|  |  | **4** | 15 | 0.7% | 53.3 | 30.9 | (36.1, 70.4) | 41 | 12 | 1.2% | 57.9 | 30.4 | (38.6, 77.2) | 54.0 | | 3 | 0.3% | 34.7 | 31.2 | (-42.8, 112.1) | 23.0 |  |
|  |  | **5** | 10 | 0.5% | 73.0 | 23.6 | (56.1, 89.9) | 81.5 | 8 | 0.8% | 69.3 | 24.9 | (48.5, 90) | 81.5 | | 2 | 0.2% | 88.0 | 11.3 | (-13.6, 189.6) | 88.0 |  |
|  | **Usual**  **activities** | **1** | 1389 | 68.8% | 80.8 | 13.8 | (80.1, 81.5) | 83 | 647 | 66.5% | 80.9 | 14.4 | (79.8, 82) | 84.0 | | 741 | 71.2% | 80.7 | 13.2 | (79.7, 81.6) | 82.0 |  |
|  |  | **2** | 385 | 19.1% | 66.1 | 18.4 | (64.3, 68) | 70 | 195 | 20.0% | 65.6 | 19.2 | (62.9, 68.3) | 70.0 | | 189 | 18.2% | 66.8 | 17.7 | (64.3, 69.3) | 70.0 |  |
|  |  | **3** | 187 | 9.3% | 54.5 | 21.2 | (51.4, 57.6) | 59 | 97 | 10.0% | 56.6 | 20.6 | (52.5, 60.8) | 60.0 | | 88 | 8.5% | 52.6 | 21.8 | (48, 57.3) | 55.0 |  |
|  |  | **4** | 46 | 2.3% | 44.7 | 23.1 | (37.8, 51.6) | 40 | 26 | 2.7% | 50.4 | 25.1 | (40.3, 60.5) | 40.0 | | 20 | 1.9% | 37.3 | 18.4 | (28.6, 45.9) | 33.0 |  |
|  |  | **5** | 11 | 0.5% | 63.7 | 28.1 | (44.9, 82.6) | 62 | 8 | 0.8% | 65.3 | 26.7 | (43, 87.5) | 71.0 | | 3 | 0.3% | 59.7 | 37.6 | (-33.6, 153) | 62.0 |  |
|  | **Pain/**  **discomfort** | **1** | 749 | 37.1% | 83.1 | 13.6 | (82.1, 84.1) | 86 | 392 | 40.3% | 83.0 | 14.4 | (81.6, 84.5) | 86.0 | | 356 | 34.2% | 83.3 | 12.6 | (82, 84.6) | 86.0 |  |
|  |  | **2** | 760 | 37.7% | 76.1 | 15.4 | (75, 77.2) | 80 | 331 | 34.0% | 74.9 | 16.2 | (73.1, 76.6) | 80.0 | | 426 | 40.9% | 77.3 | 14.4 | (75.9, 78.7) | 80.0 |  |
|  |  | **3** | 383 | 19.0% | 63.2 | 19.5 | (61.2, 65.1) | 70 | 190 | 19.5% | 62.6 | 19.9 | (59.8, 65.5) | 69.0 | | 193 | 18.5% | 63.7 | 19.2 | (61, 66.4) | 70.0 |  |
|  |  | **4** | 104 | 5.2% | 50.6 | 21.2 | (46.5, 54.7) | 50 | 49 | 5.0% | 53.8 | 21.5 | (47.6, 59.9) | 50.0 | | 55 | 5.3% | 47.7 | 20.8 | (42.1, 53.3) | 48.0 |  |
|  |  | **5** | 22 | 1.1% | 49.0 | 28.9 | (36.1, 61.8) | 41.5 | 11 | 1.1% | 54.9 | 32.3 | (33.2, 76.6) | 60.0 | | 11 | 1.1% | 43.0 | 25.3 | (26, 60) | 40.0 |  |
|  | **Anxiety/**  **depression** | **1** | 987 | 48.9% | 80.7 | 15.4 | (79.7, 81.7) | 84 | 518 | 53.2% | 80.4 | 16.3 | (79, 81.8) | 84.0 | | 469 | 45.1% | 81.1 | 14.4 | (79.8, 82.4) | 84.0 |  |
|  |  | **2** | 524 | 26.0% | 73.3 | 17.3 | (71.8, 74.8) | 78 | 232 | 23.8% | 73.2 | 16.5 | (71, 75.3) | 77.0 | | 292 | 28.1% | 73.4 | 18.0 | (71.3, 75.4) | 80.0 |  |
|  |  | **3** | 349 | 17.3% | 67.0 | 19.7 | (64.9, 69) | 71 | 148 | 15.2% | 65.9 | 20.1 | (62.6, 69.2) | 71.0 | | 199 | 19.1% | 67.9 | 19.3 | (65.2, 70.6) | 71.0 |  |
|  |  | **4** | 100 | 5.0% | 62.8 | 19.8 | (58.8, 66.7) | 70 | 44 | 4.5% | 58.3 | 21.3 | (51.8, 64.8) | 62.5 | | 55 | 5.3% | 66.8 | 17.8 | (62, 71.6) | 70.0 |  |
|  |  | **5** | 58 | 2.9% | 50.5 | 24.9 | (43.9, 57.1) | 48.5 | 31 | 3.2% | 49.9 | 23.8 | (41.1, 58.6) | 50.0 | | 26 | 2.5% | 51.6 | 27.0 | (40.7, 62.5) | 45.5 |  |

Appendix E Online index and VAS-based norms by age and general health status stratified by gender

|  |  |  | **Men** | | | | | | **Women** | | | | | |
| --- | --- | --- | --- | --- | --- | --- | --- | --- | --- | --- | --- | --- | --- | --- |
|  |  |  | **n** | **%** | **Mean** | **Standard**  **Deviation** | **95% CI** | **Median** | **n** | **%** | **Mean** | **Standard**  **Deviation** | **95% CI** | **Median** |
| **EQ-5D-5L Index** |  | **Overall** | 973 | 48.3% | 0.792 | 0.256 | (0.776, 0.808) | 0.883 | 1041 | 51.7% | 0.806 | 0.216 | (0.793, 0.819) | 0.883 |
|  | **Age** | **<25** | 57 | 5.9% | 0.855 | 0.194 | (0.804, 0.907) | 0.940 | 75 | 7.2% | 0.836 | 0.179 | (0.795, 0.877) | 0.883 |
|  |  | **25-34** | 248 | 25.5% | 0.794 | 0.291 | (0.757, 0.83) | 0.894 | 245 | 23.5% | 0.830 | 0.205 | (0.804, 0.855) | 0.883 |
|  |  | **35-44** | 201 | 20.7% | 0.782 | 0.268 | (0.745, 0.819) | 0.877 | 183 | 17.6% | 0.810 | 0.221 | (0.777, 0.842) | 0.883 |
|  |  | **45-54** | 150 | 15.4% | 0.757 | 0.266 | (0.714, 0.8) | 0.872 | 179 | 17.2% | 0.765 | 0.258 | (0.727, 0.803) | 0.844 |
|  |  | **55-64** | 156 | 16.0% | 0.774 | 0.260 | (0.733, 0.816) | 0.872 | 230 | 22.1% | 0.786 | 0.218 | (0.757, 0.814) | 0.844 |
|  |  | **65-74** | 139 | 14.3% | 0.827 | 0.174 | (0.798, 0.857) | 0.851 | 113 | 10.9% | 0.836 | 0.168 | (0.805, 0.867) | 0.883 |
|  |  | **75+** | 22 | 2.3% | 0.838 | 0.168 | (0.764, 0.913) | 0.878 | 16 | 1.5% | 0.808 | 0.169 | (0.718, 0.899) | 0.844 |
|  | **General**  **health** | **Excellent** | 147 | 15.1% | 0.846 | 0.314 | (0.795, 0.898) | 1.000 | 98 | 9.4% | 0.901 | 0.184 | (0.864, 0.938) | 0.943 |
|  |  | **Very good** | 315 | 32.4% | 0.878 | 0.193 | (0.857, 0.900) | 0.940 | 379 | 36.4% | 0.894 | 0.123 | (0.882, 0.907) | 0.940 |
|  |  | **Good** | 324 | 33.3% | 0.798 | 0.212 | (0.775, 0.821) | 0.846 | 404 | 38.8% | 0.802 | 0.173 | (0.785, 0.819) | 0.845 |
|  |  | **Fair** | 158 | 16.2% | 0.620 | 0.260 | (0.579, 0.661) | 0.641 | 131 | 12.6% | 0.591 | 0.258 | (0.547, 0.636) | 0.648 |
|  |  | **Poor** | 29 | 3.0% | 0.442 | 0.246 | (0.348, 0.536) | 0.429 | 29 | 2.8% | 0.355 | 0.327 | (0.230, 0.479) | 0.396 |
| **EQ VAS** |  | **Overall** | 973 | 48.3% | 74.5 | 19.1 | (73.3, 75.7) | 80 | 1041 | 51.7% | 74.9 | 18.3 | (73.8, 76) | 80 |
|  | **Age** | **<25** | 57 | 5.9% | 83.0 | 13.3 | (79.5, 86.5) | 86.0 | 75 | 7.2% | 77.6 | 16.6 | (73.8, 81.4) | 81.0 |
|  |  | **25-34** | 248 | 25.5% | 78.3 | 17.8 | (76, 80.5) | 83.0 | 245 | 23.5% | 77.4 | 15.6 | (75.4, 79.3) | 80.0 |
|  |  | **35-44** | 201 | 20.7% | 74.5 | 17.3 | (72, 76.9) | 80.0 | 183 | 17.6% | 75.2 | 17.4 | (72.6, 77.7) | 80.0 |
|  |  | **45-54** | 150 | 15.4% | 70.2 | 20.5 | (66.9, 73.5) | 76.5 | 179 | 17.2% | 72.1 | 18.9 | (69.3, 74.9) | 79.0 |
|  |  | **55-64** | 156 | 16.0% | 71.3 | 21.4 | (67.9, 74.7) | 76.5 | 230 | 22.1% | 71.6 | 21.6 | (68.8, 74.4) | 79.0 |
|  |  | **65-74** | 139 | 14.3% | 72.6 | 19.4 | (69.3, 75.8) | 78.0 | 113 | 10.9% | 77.4 | 17.3 | (74.2, 80.6) | 81.0 |
|  |  | **75+** | 22 | 2.3% | 74.1 | 21.8 | (64.5, 83.8) | 80.0 | 16 | 1.5% | 82.2 | 8.5 | (77.6, 86.7) | 82.5 |
|  | **General**  **health** | **Excellent** | 147 | 15.1% | 88.0 | 14.3 | (85.6, 90.3) | 91.0 | 98 | 9.4% | 89.1 | 13.1 | (86.4, 91.7) | 92.5 |
|  |  | **Very good** | 315 | 32.4% | 84.5 | 9.1 | (83.4, 85.5) | 85.0 | 379 | 36.4% | 83.9 | 10.1 | (82.9, 84.9) | 85.0 |
|  |  | **Good** | 324 | 33.3% | 73.4 | 12.1 | (72.1, 74.8) | 75.0 | 404 | 38.8% | 72.9 | 13.8 | (71.6, 74.2) | 75.0 |
|  |  | **Fair** | 158 | 16.2% | 51.8 | 18.5 | (48.9, 54.7) | 53.0 | 131 | 12.6% | 53.7 | 18.6 | (50.5, 56.9) | 55.0 |
|  |  | **Poor** | 29 | 3.0% | 33.0 | 18.6 | (26.0, 40.1) | 30.0 | 29 | 2.8% | 32.7 | 17.8 | (26.0, 39.5) | 30.0 |

Appendix F US online sample frequency of self-reported problems by dimension-level and gender

|  |  | **Complete Online sample** | | | | | | | | | | | | | | **Online male sample** | | | | | | | | | | | | | | **Online female sample** | | | | | | | | | | | | | |
| --- | --- | --- | --- | --- | --- | --- | --- | --- | --- | --- | --- | --- | --- | --- | --- | --- | --- | --- | --- | --- | --- | --- | --- | --- | --- | --- | --- | --- | --- | --- | --- | --- | --- | --- | --- | --- | --- | --- | --- | --- | --- | --- | --- |
| **Age band** | | **<25** | | **25-34** | | **35-44** | | **45-54** | | **55-64** | | **65-74** | | **75+** | | **<25** | | **25-34** | | **35-44** | | **45-54** | | **55-64** | | **65-74** | | **75+** | | **<25** | | **25-34** | | **35-44** | | **45-54** | | **55-64** | | **65-74** | | **75+** | |
| **MO** | **1** | 110 | 82.7% | 398 | 80.6% | 294 | 76.4% | 213 | 64.6% | 233 | 60.4% | 157 | 62.3% | 20 | 52.6% | 46 | 80.7% | 185 | 74.6% | 144 | 71.6% | 90 | 60.0% | 99 | 63.5% | 84 | 60.4% | 13 | 59.1% | 63 | 84.0% | 212 | 86.5% | 149 | 81.4% | 123 | 68.7% | 134 | 58.3% | 73 | 64.6% | 7 | 43.8% |
|  | **2** | 16 | 12.0% | 52 | 10.5% | 52 | 13.5% | 67 | 20.3% | 100 | 25.9% | 60 | 23.8% | 14 | 36.8% | 6 | 10.5% | 30 | 12.1% | 31 | 15.4% | 34 | 22.7% | 34 | 21.8% | 34 | 24.5% | 7 | 31.8% | 10 | 13.3% | 22 | 9.0% | 21 | 11.5% | 32 | 17.9% | 66 | 28.7% | 26 | 23.0% | 7 | 43.8% |
|  | **3** | 6 | 4.5% | 34 | 6.9% | 30 | 7.8% | 31 | 9.4% | 39 | 10.1% | 29 | 11.5% | 3 | 7.9% | 4 | 7.0% | 25 | 10.1% | 20 | 10.0% | 18 | 12.0% | 14 | 9.0% | 18 | 13.0% | 1 | 4.6% | 2 | 2.7% | 9 | 3.7% | 10 | 5.5% | 13 | 7.3% | 25 | 10.9% | 11 | 9.7% | 2 | 12.5% |
|  | **4** | 0 | 0.0% | 8 | 1.6% | 4 | 1.0% | 14 | 4.2% | 11 | 2.9% | 4 | 1.6% | 1 | 2.6% | 0 | 0.0% | 6 | 2.4% | 2 | 1.0% | 5 | 3.3% | 8 | 5.1% | 2 | 1.4% | 1 | 4.6% | 0 | 0.0% | 2 | 0.8% | 2 | 1.1% | 9 | 5.0% | 3 | 1.3% | 2 | 1.8% | 0 | 0.0% |
|  | **5** | 1 | 0.8% | 2 | 0.4% | 5 | 1.3% | 5 | 1.5% | 3 | 0.8% | 2 | 0.8% | 0 | 0.0% | 1 | 1.8% | 2 | 0.8% | 4 | 2.0% | 3 | 2.0% | 1 | 0.6% | 1 | 0.7% | 0 | 0.0% | 0 | 0.0% | 0 | 0.0% | 1 | 0.6% | 2 | 1.1% | 2 | 0.9% | 1 | 0.9% | 0 | 0.0% |
| **SC** | **1** | 120 | 90.2% | 436 | 88.3% | 324 | 84.2% | 278 | 84.2% | 336 | 87.1% | 226 | 89.7% | 35 | 92.1% | 49 | 86.0% | 206 | 83.1% | 160 | 79.6% | 120 | 80.0% | 128 | 82.1% | 120 | 86.3% | 20 | 90.9% | 70 | 93.3% | 229 | 93.5% | 164 | 89.6% | 157 | 87.7% | 208 | 90.4% | 106 | 93.8% | 15 | 93.8% |
|  | **2** | 10 | 7.5% | 28 | 5.7% | 41 | 10.7% | 32 | 9.7% | 40 | 10.4% | 20 | 7.9% | 2 | 5.3% | 5 | 8.8% | 19 | 7.7% | 25 | 12.4% | 16 | 10.7% | 22 | 14.1% | 14 | 10.1% | 1 | 4.6% | 5 | 6.7% | 9 | 3.7% | 15 | 8.2% | 16 | 8.9% | 18 | 7.8% | 6 | 5.3% | 1 | 6.3% |
|  | **3** | 3 | 2.3% | 22 | 4.5% | 12 | 3.1% | 15 | 4.6% | 7 | 1.8% | 5 | 2.0% | 1 | 2.6% | 3 | 5.3% | 16 | 6.5% | 10 | 5.0% | 10 | 6.7% | 4 | 2.6% | 4 | 2.9% | 1 | 4.6% | 0 | 0.0% | 6 | 2.5% | 2 | 1.1% | 5 | 2.8% | 3 | 1.3% | 1 | 0.9% | 0 | 0.0% |
|  | **4** | 0 | 0.0% | 4 | 0.8% | 3 | 0.8% | 4 | 1.2% | 3 | 0.8% | 1 | 0.4% | 0 | 0.0% | 0 | 0.0% | 4 | 1.6% | 2 | 1.0% | 3 | 2.0% | 2 | 1.3% | 1 | 0.7% | 0 | 0.0% | 0 | 0.0% | 0 | 0.0% | 1 | 0.6% | 1 | 0.6% | 1 | 0.4% | 0 | 0.0% | 0 | 0.0% |
|  | **5** | 0 | 0.0% | 4 | 0.8% | 5 | 1.3% | 1 | 0.3% | 0 | 0.0% | 0 | 0.0% | 0 | 0.0% | 0 | 0.0% | 3 | 1.2% | 4 | 2.0% | 1 | 0.7% | 0 | 0.0% | 0 | 0.0% | 0 | 0.0% | 0 | 0.0% | 1 | 0.4% | 1 | 0.6% | 0 | 0.0% | 0 | 0.0% | 0 | 0.0% | 0 | 0.0% |
| **UA** | **1** | 106 | 79.7% | 378 | 76.5% | 276 | 71.7% | 207 | 62.7% | 229 | 59.3% | 169 | 67.1% | 24 | 63.2% | 45 | 79.0% | 181 | 73.0% | 136 | 67.7% | 89 | 59.3% | 92 | 59.0% | 90 | 64.8% | 14 | 63.6% | 60 | 80.0% | 197 | 80.4% | 140 | 76.5% | 118 | 65.9% | 137 | 59.6% | 79 | 69.9% | 10 | 62.5% |
|  | **2** | 14 | 10.5% | 61 | 12.4% | 66 | 17.1% | 70 | 21.2% | 104 | 26.9% | 60 | 23.8% | 10 | 26.3% | 6 | 10.5% | 31 | 12.5% | 39 | 19.4% | 33 | 22.0% | 42 | 26.9% | 38 | 27.3% | 6 | 27.3% | 8 | 10.7% | 30 | 12.2% | 27 | 14.8% | 36 | 20.1% | 62 | 27.0% | 22 | 19.5% | 4 | 25.0% |
|  | **3** | 11 | 8.3% | 41 | 8.3% | 33 | 8.6% | 38 | 11.5% | 41 | 10.6% | 19 | 7.5% | 4 | 10.5% | 5 | 8.8% | 27 | 10.9% | 19 | 9.5% | 20 | 13.3% | 15 | 9.6% | 9 | 6.5% | 2 | 9.1% | 6 | 8.0% | 13 | 5.3% | 13 | 7.1% | 18 | 10.1% | 26 | 11.3% | 10 | 8.9% | 2 | 12.5% |
|  | **4** | 2 | 1.5% | 11 | 2.2% | 7 | 1.8% | 14 | 4.2% | 9 | 2.3% | 3 | 1.2% | 0 | 0.0% | 1 | 1.8% | 7 | 2.8% | 4 | 2.0% | 7 | 4.7% | 5 | 3.2% | 2 | 1.4% | 0 | 0.0% | 1 | 1.3% | 4 | 1.6% | 3 | 1.6% | 7 | 3.9% | 4 | 1.7% | 1 | 0.9% | 0 | 0.0% |
|  | **5** | 0 | 0.0% | 3 | 0.6% | 3 | 0.8% | 1 | 0.3% | 3 | 0.8% | 1 | 0.4% | 0 | 0.0% | 0 | 0.0% | 2 | 0.8% | 3 | 1.5% | 1 | 0.7% | 2 | 1.3% | 0 | 0.0% | 0 | 0.0% | 0 | 0.0% | 1 | 0.4% | 0 | 0.0% | 0 | 0.0% | 1 | 0.4% | 1 | 0.9% | 0 | 0.0% |
| **PD** | **1** | 73 | 54.9% | 260 | 52.6% | 135 | 35.1% | 89 | 27.0% | 108 | 28.0% | 73 | 29.0% | 11 | 29.0% | 34 | 59.7% | 138 | 55.7% | 74 | 36.8% | 47 | 31.3% | 50 | 32.1% | 42 | 30.2% | 7 | 31.8% | 39 | 52.0% | 122 | 49.8% | 60 | 32.8% | 42 | 23.5% | 58 | 25.2% | 31 | 27.4% | 4 | 25.0% |
|  | **2** | 45 | 33.8% | 151 | 30.6% | 158 | 41.0% | 120 | 36.4% | 158 | 40.9% | 111 | 44.1% | 17 | 44.7% | 16 | 28.1% | 62 | 25.0% | 74 | 36.8% | 47 | 31.3% | 59 | 37.8% | 63 | 45.3% | 10 | 45.5% | 28 | 37.3% | 88 | 35.9% | 84 | 45.9% | 72 | 40.2% | 99 | 43.0% | 48 | 42.5% | 7 | 43.8% |
|  | **3** | 12 | 9.0% | 59 | 11.9% | 69 | 17.9% | 89 | 27.0% | 87 | 22.5% | 59 | 23.4% | 8 | 21.1% | 6 | 10.5% | 33 | 13.3% | 41 | 20.4% | 43 | 28.7% | 34 | 21.8% | 29 | 20.9% | 4 | 18.2% | 6 | 8.0% | 26 | 10.6% | 28 | 15.3% | 46 | 25.7% | 53 | 23.0% | 30 | 26.6% | 4 | 25.0% |
|  | **4** | 3 | 2.3% | 17 | 3.4% | 20 | 5.2% | 27 | 8.2% | 27 | 7.0% | 9 | 3.6% | 1 | 2.6% | 1 | 1.8% | 10 | 4.0% | 9 | 4.5% | 13 | 8.7% | 10 | 6.4% | 5 | 3.6% | 1 | 4.6% | 2 | 2.7% | 7 | 2.9% | 11 | 6.0% | 14 | 7.8% | 17 | 7.4% | 4 | 3.5% | 0 | 0.0% |
|  | **5** | 0 | 0.0% | 7 | 1.4% | 3 | 0.8% | 5 | 1.5% | 6 | 1.6% | 0 | 0.0% | 1 | 2.6% | 0 | 0.0% | 5 | 2.0% | 3 | 1.5% | 0 | 0.0% | 3 | 1.9% | 0 | 0.0% | 0 | 0.0% | 0 | 0.0% | 2 | 0.8% | 0 | 0.0% | 5 | 2.8% | 3 | 1.3% | 0 | 0.0% | 1 | 6.3% |
| **AD** | **1** | 57 | 42.9% | 197 | 39.9% | 161 | 41.8% | 153 | 46.4% | 214 | 55.4% | 176 | 69.8% | 29 | 76.3% | 32 | 56.1% | 113 | 45.6% | 85 | 42.3% | 77 | 51.3% | 90 | 57.7% | 104 | 74.8% | 17 | 77.3% | 25 | 33.3% | 84 | 34.3% | 76 | 41.5% | 76 | 42.5% | 124 | 53.9% | 72 | 63.7% | 12 | 75.0% |
|  | **2** | 33 | 24.8% | 126 | 25.5% | 114 | 29.6% | 96 | 29.1% | 97 | 25.1% | 53 | 21.0% | 5 | 13.2% | 11 | 19.3% | 59 | 23.8% | 60 | 29.9% | 41 | 27.3% | 34 | 21.8% | 24 | 17.3% | 3 | 13.6% | 22 | 29.3% | 67 | 27.4% | 54 | 29.5% | 55 | 30.7% | 63 | 27.4% | 29 | 25.7% | 2 | 12.5% |
|  | **3** | 30 | 22.6% | 108 | 21.9% | 73 | 19.0% | 60 | 18.2% | 56 | 14.5% | 18 | 7.1% | 4 | 10.5% | 10 | 17.5% | 43 | 17.3% | 42 | 20.9% | 23 | 15.3% | 22 | 14.1% | 6 | 4.3% | 2 | 9.1% | 19 | 25.3% | 64 | 26.1% | 31 | 16.9% | 37 | 20.7% | 34 | 14.8% | 12 | 10.6% | 2 | 12.5% |
|  | **4** | 8 | 6.0% | 41 | 8.3% | 24 | 6.2% | 12 | 3.6% | 11 | 2.9% | 4 | 1.6% | 0 | 0.0% | 3 | 5.3% | 20 | 8.1% | 6 | 3.0% | 5 | 3.3% | 6 | 3.9% | 4 | 2.9% | 0 | 0.0% | 5 | 6.7% | 21 | 8.6% | 17 | 9.3% | 7 | 3.9% | 5 | 2.2% | 0 | 0.0% | 0 | 0.0% |
|  | **5** | 5 | 3.8% | 22 | 4.5% | 13 | 3.4% | 9 | 2.7% | 8 | 2.1% | 1 | 0.4% | 0 | 0.0% | 1 | 1.8% | 13 | 5.2% | 8 | 4.0% | 4 | 2.7% | 4 | 2.6% | 1 | 0.7% | 0 | 0.0% | 4 | 5.3% | 9 | 3.7% | 5 | 2.7% | 4 | 2.2% | 4 | 1.7% | 0 | 0.0% | 0 | 0.0% |

Appendix G Comparison of online and face-to-face respondents to the US general population

|  | US population  % (2016) | Face-to-face  respondents  N=223 | | Online  respondents  N=290 | |
| --- | --- | --- | --- | --- | --- |
| Characteristic |  | N | % | N | % |
| Age band[1] |  |  |  |  |  |
| 65-74 | 58.3% | 127 | 57.0% | 252 | 86.9% |
| 75+ | 41.7% | 96 | 43.0% | 38 | 13.1% |
| Race[1] |  |  |  |  |  |
| Black | 8.9% | 28 | 12.6% | 30 | 10.3% |
| White | 77.3% | 169 | 75.8% | 242 | 83.5% |
| Ethnicity[1] |  |  |  |  |  |
| Hispanic | 8.0% | 22 | 9.9% | 26 | 9.0% |
| Gender[1] |  |  |  |  |  |
| Female | 55.8% | 118 | 52.9% | 129 | 44.5% |
| Male | 44.2% | 105 | 47.1% | 161 | 55.5% |
| Educational attainment - male*[1] |  |  |  |  |  |
| Less than high school | 15.6% | 5 | 4.8% | 1 | 0.6% |
| High school graduate | 27.3% | 33 | 31.4% | 38 | 23.6% |
| Some college or Associate degree** | 25.0% | 20 | 19.1% | 51 | 31.7% |
| Bachelor’s degree or higher | 32.1% | 47 | 44.8% | 71 | 44.1% |
| Educational attainment - female*[1] |  |  |  |  |  |
| Less than high school | 17.2% | 8 | 6.8% | 2 | 1.6% |
| High school graduate | 34.9% | 41 | 35.0% | 38 | 29.5% |
| Some college or Associate degree** | 25.6% | 25 | 21.4% | 42 | 32.6% |
| Bachelor’s degree or higher | 22.3% | 43 | 36.8% | 47 | 36.4% |
| General health[2] |  |  |  |  |  |
| Excellent | 9.9% | 28 | 12.6% | 14 | 4.8% |
| Very good | 28.7% | 87 | 39.2% | 94 | 32.4% |
| Good | 37.4% | 70 | 31.5% | 122 | 42.1% |
| Fair | 19.5% | 32 | 14.4% | 51 | 17.6% |
| Poor | 4.5% | 5 | 2.3% | 9 | 3.1% |

*Educational attainment only reported by gender in reference

**Some college or associate degree and technical school attendance were separately reported in collected data; these 2 categories were combined for comparison to US general population

1. Roberts, A. W., Ogunwole, S. U., Blakeslee, L., & Rabe, M. A. (2018). The Population 65 Years and Older in the United States: 2016. <https://www.census.gov/content/dam/Census/library/publications/2018/acs/ACS-38.pdf>.

2. Agency for Healthcare Research and Quality (2017). 2017 Medical Expenditure Panel Survey. <https://www.meps.ahrq.gov/mepsweb/data_stats/download_data_files_detail.jsp?cboPufNumber=HC-201>. Accessed August 9 2020.
